# Broad transcriptomic dysregulation occurs across the cerebral cortex in ASD

**DOI:** 10.1038/s41586-022-05377-7

**Published:** 2022-11-02

**Authors:** Michael J. Gandal, Jillian R. Haney, Brie Wamsley, Chloe X. Yap, Sepideh Parhami, Prashant S. Emani, Nathan Chang, George T. Chen, Gil D. Hoftman, Diego de Alba, Gokul Ramaswami, Christopher L. Hartl, Arjun Bhattacharya, Chongyuan Luo, Ting Jin, Daifeng Wang, Riki Kawaguchi, Diana Quintero, Jing Ou, Ye Emily Wu, Neelroop N. Parikshak, Vivek Swarup, T. Grant Belgard, Mark Gerstein, Bogdan Pasaniuc, Daniel H. Geschwind

**Affiliations:** 1 Center for Neurobehavioral Genetics, Semel Institute for Neuroscience and Human Behavior, David Geffen School of Medicine, University of California, Los Angeles, CA USA; 2 Center for Autism Research and Treatment, Semel Institute of Neuroscience and Human Behavior, University of California, Los Angeles, CA USA; 3 Department of Psychiatry and Biobehavioral Sciences, David Geffen School of Medicine, University of California, Los Angeles, CA USA; 4 Department of Human Genetics, David Geffen School of Medicine, University of California, Los Angeles, CA USA; 5 Department of Neurology, David Geffen School of Medicine, University of California, Los Angeles, CA USA; 6 Mater Research Institute, University of Queensland, Brisbane, Queensland Australia; 7 Institute for Molecular Biosciences, University of Queensland, Brisbane, Queensland Australia; 8 Computational Biology & Bioinformatics Program, Yale University, New Haven, CT USA; 9 Department of Pathology and Laboratory Medicine, David Geffen School of Medicine, University of California, Los Angeles, CA USA; 10 Waisman Center and Department of Biostatistics and Medical Informatics, University of Wisconsin—Madison, Madison, WI USA; 11 Institute for Memory Impairments and Neurological Disorders, University of California, Irvine, CA USA; 12 The Bioinformatics CRO, Niceville, FL USA; 13 Present Address: Lifespan Brain Institute at Penn Medicine and The Children’s Hospital of Philadelphia, Department of Psychiatry, University of Pennsylvania, Philadelphia, PA USA

**Keywords:** Autism spectrum disorders, Transcriptomics, Genetics of the nervous system, RNA sequencing, Gene regulatory networks

## Abstract

Neuropsychiatric disorders classically lack defining brain pathologies, but recent work has demonstrated dysregulation at the molecular level, characterized by transcriptomic and epigenetic alterations^1–3^. In autism spectrum disorder (ASD), this molecular pathology involves the upregulation of microglial, astrocyte and neural–immune genes, the downregulation of synaptic genes, and attenuation of gene-expression gradients in cortex^1,2,4–6^. However, whether these changes are limited to cortical association regions or are more widespread remains unknown. To address this issue, we performed RNA-sequencing analysis of 725 brain samples spanning 11 cortical areas from 112 post-mortem samples from individuals with ASD and neurotypical controls. We find widespread transcriptomic changes across the cortex in ASD, exhibiting an anterior-to-posterior gradient, with the greatest differences in primary visual cortex, coincident with an attenuation of the typical transcriptomic differences between cortical regions. Single-nucleus RNA-sequencing and methylation profiling demonstrate that this robust molecular signature reflects changes in cell-type-specific gene expression, particularly affecting excitatory neurons and glia. Both rare and common ASD-associated genetic variation converge within a downregulated co-expression module involving synaptic signalling, and common variation alone is enriched within a module of upregulated protein chaperone genes. These results highlight widespread molecular changes across the cerebral cortex in ASD, extending beyond association cortex to broadly involve primary sensory regions.

## Abstract (web summary)

RNA sequencing reveals widespread transcriptomic changes across the cerebral cortex in autism spectrum disorder, including primary sensory regions, in addition to association regions, as well as an attenuation of regional identity.

## Main

Similar to other neuropsychiatric disorders, the risk factors for ASD involve a substantial genetic component, which is profoundly complex and multifactorial, involving hundreds of risk genes^7,8^. Yet, despite aetiological heterogeneity, molecular profiling studies in ASD have found consistent patterns of transcriptomic and epigenetic dysregulation involving frontal and temporal cortex in the majority of cases^1–5,9^. Whether this represents a focal, regional or more generalized molecular pathology is not known. Understanding the nature and distribution of these molecular changes is essential to deciphering the neurobiological underpinnings of ASD.

## Cortex-wide transcriptomic changes in ASD

**Fig. 1:**
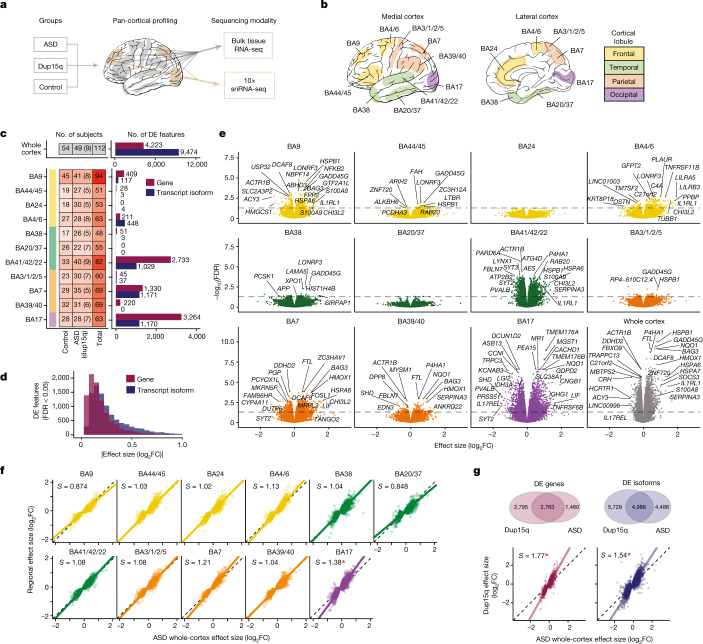
ASD-associated transcriptomic differences across 11 cortical regions. **a**, Study experimental design. Eleven regions were profiled by bulk RNA-seq, and three regions were also profiled by single-nucleus RNA sequencing (snRNA-seq) (Fig. 4). **b**, Human cortical Brodmann areas (BA) sequenced, with cortical lobules coloured consistently throughout this figure. **c**, The number of unique individuals by region and diagnosis (left), with the number of additional individuals with dup15q syndrome included in parentheses. Right, the number of differentially expressed (DE) (linear mixed model FDR < 0.05) features (genes and transcript isoforms) across the whole cortex (top) or within individual regions (below). **d**, Absolute value of effect-size changes are shown for differentially expressed genes and isoforms for whole-cortex analyses. Larger effect-size changes are observed at the transcript isoform level. **e**, Volcano plots show the top differentially expressed genes within regions and across the whole cortex. **f**, Differential expression effect size (log_2_FC) of individual regions compared with whole-cortex changes for the 4,223 cortex-wide differentially expressed genes. Slope (*S*) is calculated using principal components regression, with *P < 0.05 for *S* significantly different from unity by bootstrap (Methods). **g**, Top, Venn diagrams depicting the number of genes and isoforms that were differentially expressed across the whole cortex in dup15q samples compared with idiopathic ASD. Bottom, idiopathic ASD whole-cortex log_2_FC compared to the dup15q whole-cortex log_2_FC for the ASD whole-cortex differentially expressed genes (left) and isoforms (right). Slope is calculated using principal components regression.

**Extended Data Fig. 1:**
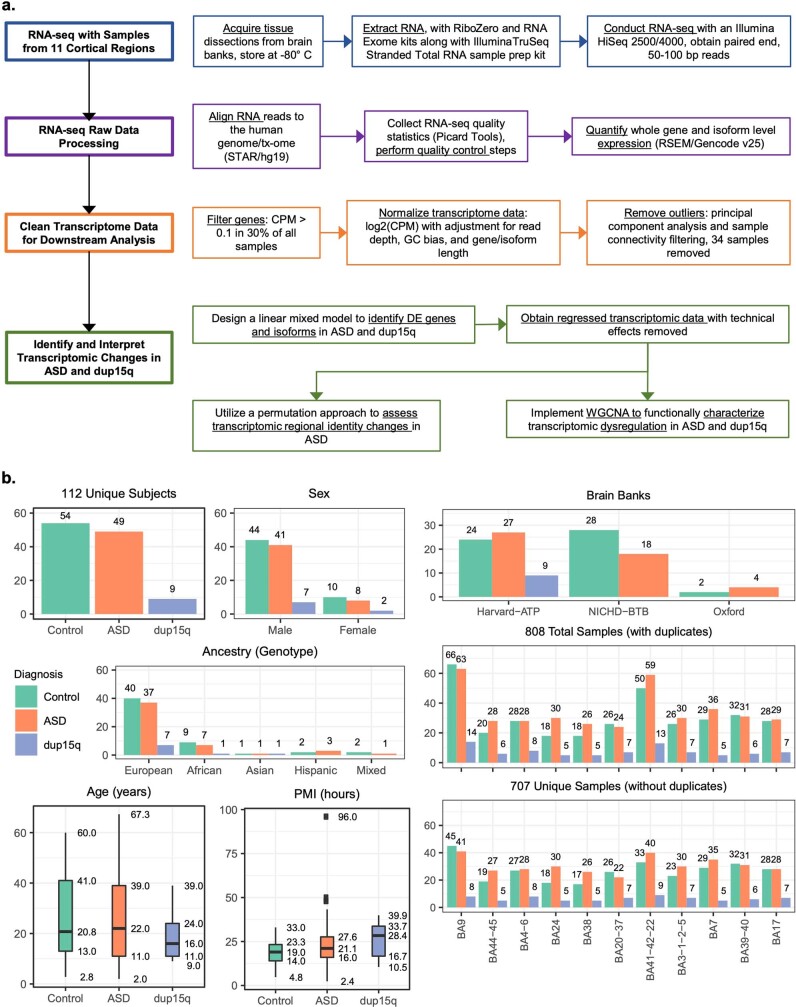
Experiment Workflow and Sample Overview. a. Overview of experiment workflow. b. Summary of sample composition (biological data, brain bank source, and PMI).

**Extended Data Fig. 2:**
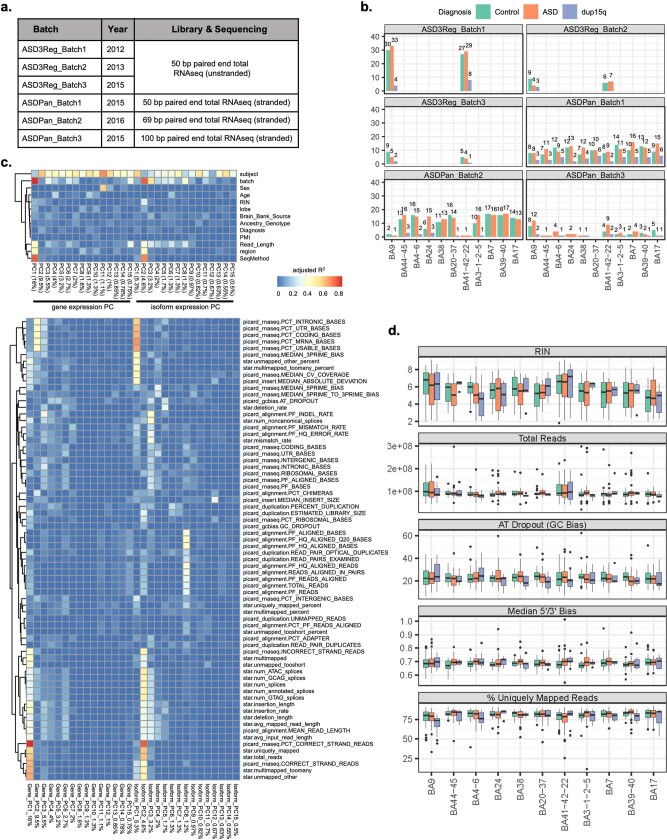
Quality Control Measures. a. Sequencing batches. b. Brain region and case-control sample sizes across batches. c. Top 15 expression PCs (gene and transcript, with % of variance explained denoted) association with meta data (top) and sequencing statistics (bottom). d. Boxplots show median and interquartile range (25^th^/75^th^ percentile; bounds of box) +/− 1.5 times the interquartile range (whiskers) for RIN values and sequencing statistics across diagnoses x regions, with the number of biologically independently samples per region and diagnosis as indicated in Fig. 1.

**Extended Data Fig. 3:**
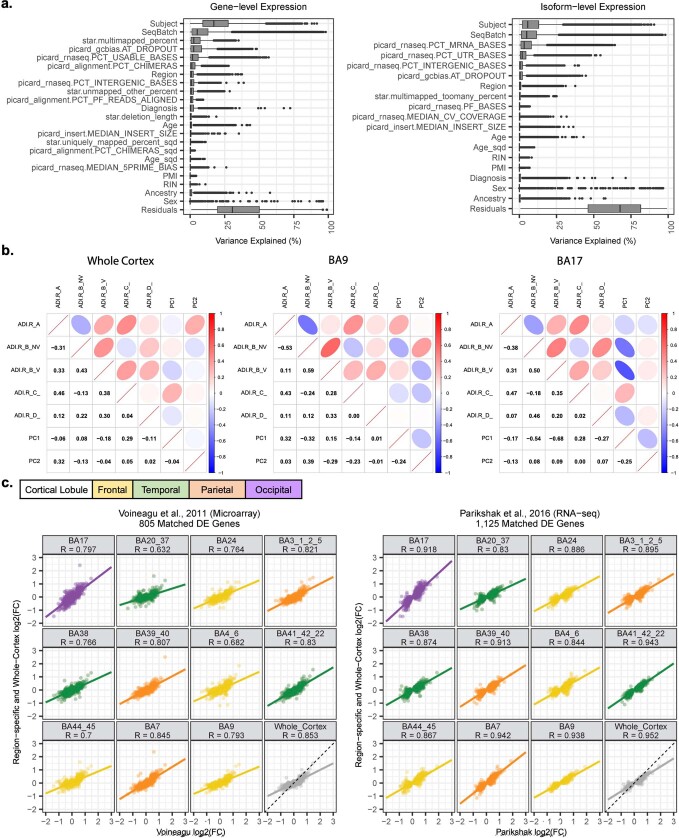
Model Covariates, ADI-R Correlations, and Previous Studies Across 11 Cortical Regions. a. For the covariates selected for the gene (left) and transcript (right) linear mixed models, % of expression variance explained across all genes/transcripts. Boxplots show median and interquartile range (25^th^/75^th^ percentile; bounds of box) +/− 1.5 times the interquartile range (whiskers) for 24,836 and 99,819 distinct genes and isoforms, respectively, measured across 725 brain samples from 112 unique subjects. b. Spearman correlations for available ADI-R scores with the first and second principal components of ASD differentially expressed genes, calculated across all regions (whole cortex), BA9, and BA17. c. For the Voineagu et al. and Parikshak et al. studies^4,5^, the ASD log_2_FC of differentially expressed genes identified in these studies, compared to this dataset (Spearman’s correlation rho, R, is plotted along with the linear least squares regression best fit line).

**Extended Data Fig. 4:**
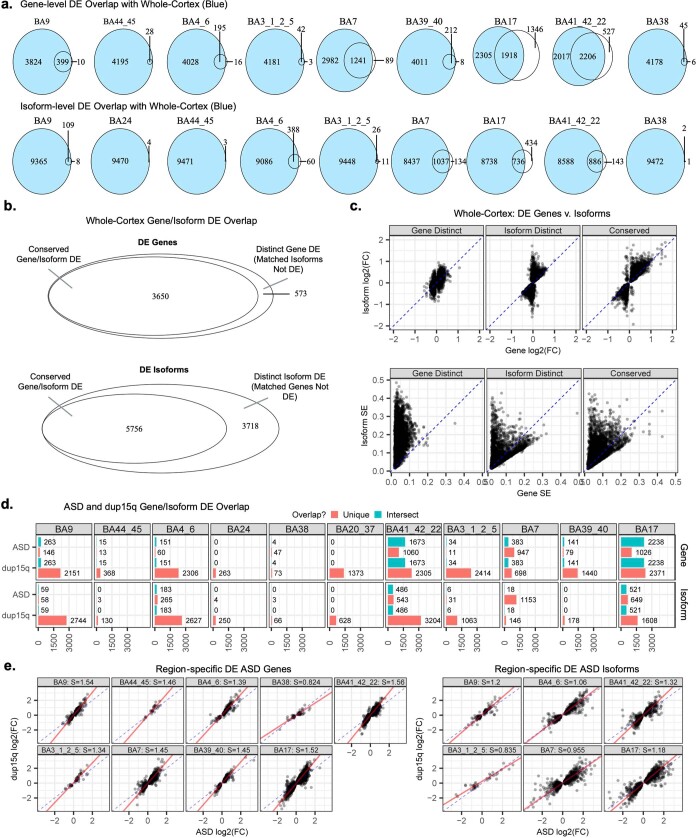
Transcriptomic Changes Across 11 Cortical Regions. a. Overlap of Whole-Cortex differentially expressed ASD genes and transcripts (blue) with other cortical region differentially expressed genes (no colour). Regions with no third numeric label on the right completely overlap with the Whole-Cortex differentially expressed genes. b. For the Whole-Cortex differential expression, overlap of genes and transcripts. Regions not shown have no unique differential expression. c. log_2_FC (top) and standard error (SE, bottom) of the Whole-Cortex ASD differential expression overlapping and distinct genes and transcripts. d. Overlap in differential expression for ASD and dup15q genes and transcripts. e. For regions with differentially expressed ASD genes (left) and transcripts (right), ASD log_2_FC v. dup15q log_2_FC for specific regions (with principal components regression slope, S).

Here, we conducted RNA-sequencing analysis (RNA-seq) to identify gene and transcript (alternatively spliced gene isoforms) changes across 725 samples spanning 11 distinct brain regions and all four cortical lobules (frontal, parietal, temporal and occipital), including multiple association and primary sensory areas, from 49 individuals with idiopathic ASD and 54 matched neurotypical controls (Fig. 1a–c, Extended Data Figs. 1–3, Supplementary Data 1 and  Methods). Compared with previous work^4,5^, this represents a more than threefold increase in the number of samples and regions profiled. In line with the increased statistical power, we found 4,223 genes and 9,474 transcripts (false discovery rate (FDR) < 0.05) that were differentially expressed cortex-wide, a notable increase compared with previous analyses^1,5^ (Fig. 1c, Extended Data Figs. 3 and 4 and Supplementary Data 3). We identified distinct differential expression signals in transcripts than at the gene level, with transcript changes exhibiting a greater magnitude of effect in ASD than their matched genes (Fig. 1d, Extended Data Fig. 4 and, Supplementary Data 2), supporting a substantial role for alternative splicing and isoform expression in ASD, consistent with partitioning of common variant heritability^10^ and previous transcriptomic analyses^1^.

We next sought to determine the regional consistency of these patterns by calculating differential expression separately within each cortical region and comparing regional effect-size changes (log_2_ fold change (FC)) with the corresponding whole-cortex signature (Fig. 1e,f, Extended Data Fig. 3, Supplementary Data 2 and Methods). Although the number of differentially expressed genes varies considerably, which may reflect sample size differences **(**Fig. 1c), we observe consistent transcriptomic signatures of ASD across all 11 cortical regions profiled, with highly concordant effect-size changes across each region compared to the whole-cortex signature (Fig. 1f). We observed the greatest signal in the primary visual cortex (BA17), with 3,264 differentially expressed genes, of which 59% overlapped with those observed globally (Fig. 1c,e, Extended Data Fig. 4 and Supplementary Data 2). Additionally, effect-size changes were significantly greater in BA17 compared with the whole-cortex signal, which was not observed for any of the other regions assessed (Fig. 1f and Methods). Together, these results demonstrate a consistent cortex-wide transcriptomic signature of ASD that is most pronounced posteriorly in BA17.

We next evaluated differential gene and transcript expression in an additional 83 pan-cortical samples from 9 subjects with maternal dup15q syndrome, a rare genetic disorder characterized by duplications in the chromosomal region 15q11–q13. Dup15q syndrome is one of the most common forms of syndromic ASD, and gene-expression changes in these subjects was previously shown to strongly parallel genetic changes in idiopathic ASD in frontal and temporal cortex, but with a greater magnitude of effect^5^. We replicated these previous results broadly across the cortical regions examined, finding substantial overlap in transcriptomic changes between dup15q and idiopathic ASD and with dup15q exhibiting a greater magnitude of gene-expression dysregulation overall (Fig. 1g, Extended Data Fig. 4 and Supplementary Data 2). BA17 also exhibited the greatest number of differentially expressed genes in dup15q (Extended Data Fig. 4). These results demonstrate that the molecular pathology shared by this rare genetic form of ASD and idiopathic ASD is widespread across distinct regions of the cortex, and that some commonalities in regional variance of effect exist, with both conditions affecting sensory areas in addition to higher-order association areas.

## Attenuation of regional identity

In the neurotypical brain, cortical regions can be distinguished on the basis of differences in gene expression, which primarily reflect variability in the cytoarchitecture, connectivity, and function of each region—with V1 being the most distinct^11–14^. We previously observed a marked attenuation of these typical gene-expression differences between two regions—frontal and temporal lobe—in ASD^4,5^, which we refer to here as an attenuation of transcriptomic regional identity (ARI). This significant reduction in the magnitude of gene-expression differences between these two cortical association regions suggested an alteration in their developmental patterning, connectivity, and/or ongoing functioning in ASD. Here, we sought to understand the extent of these molecular alterations to determine whether they were indeed focal or more widespread, involving additional association or sensory regions.

**Fig. 2:**
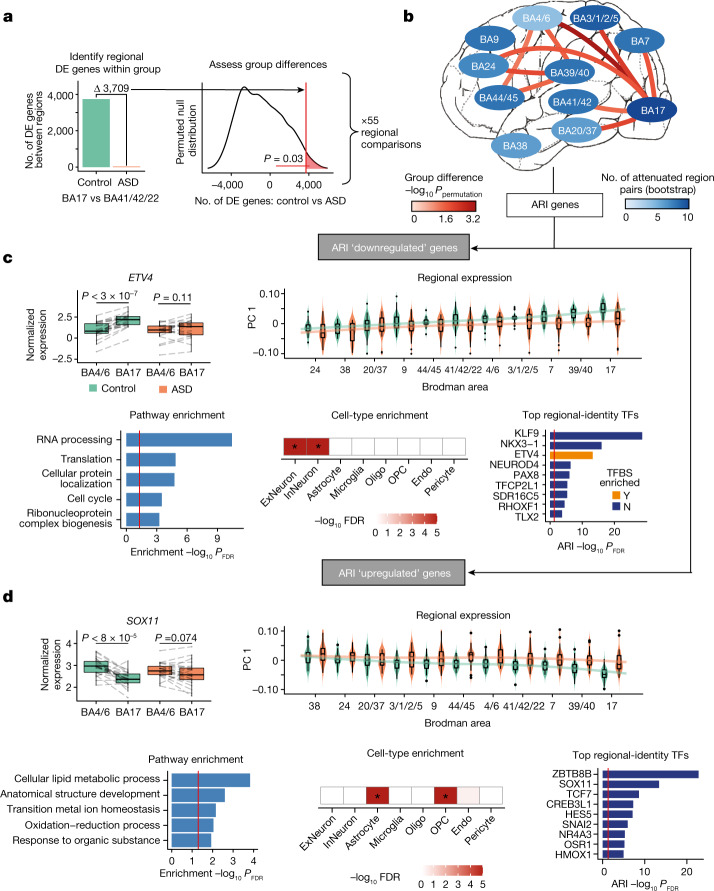
Cortex-wide transcriptomic regional-identity attenuation in ASD. **a**, Overview of methods for identifying statistically significant differences in transcriptomic regional identity in ASD. The regional comparison of BA17 versus BA41/42/22 is used here as an example. Left, the number of differentially expressed genes between regions is calculated in controls and ASD samples. Right, a permuted null distribution is then used to determine the significance of the difference in differentially expressed genes between controls and ASD samples. **b**, Regional comparisons of ARI in ASD, with those comparisons reaching a permutation *P* < 0.05 connected with a line (red scale). Cortex-wide attenuation identified with a bootstrap approach (Methods) is summarized by region colour (blue scale; 0, no pairs exhibiting attenuation in ASD; 10, all pairs exhibit attenuation in ASD). For region colour, a regional pair is considered attenuated if it contains less differential expression in ASD compared with controls. ARI genes are extracted from regional comparisons with permutation *P* < 0.05 (Methods). **c**,**d**, Overview of downregulated (**c**) and upregulated (**d**) ARI genes. Top left, attenuated transcription factors (TFs) in BA17 and BA4/6. Lines link paired samples from the same individual, and the paired Wilcoxon signed-rank test *P-*value is plotted above the box plots. Top right, principal component 1 (PC 1) of ARI genes across all regions; regions are ordered by the control median. Bottom left and bottom centre, gene ontology and cell-type enrichment (*FDR < 0.05), respectively. Bottom right, top 10 attenuated transcription factors; FDR is representative of how well these transcription factors distinguish BA17 and BA39/40 from the remaining nine other cortical regions in controls (Methods). Enrichment for transcription factor binding sites is also depicted (Bonferroni-corrected *P-*value < 0.05 is required for enrichment). ExNeuron, excitatory neuron; InNeuron, inhibitory neuron; oligo, oligodendrocyte; OPC, oligodendrocyte precursor cell; endo, endothelial cell.

**Extended Data Fig. 5:**
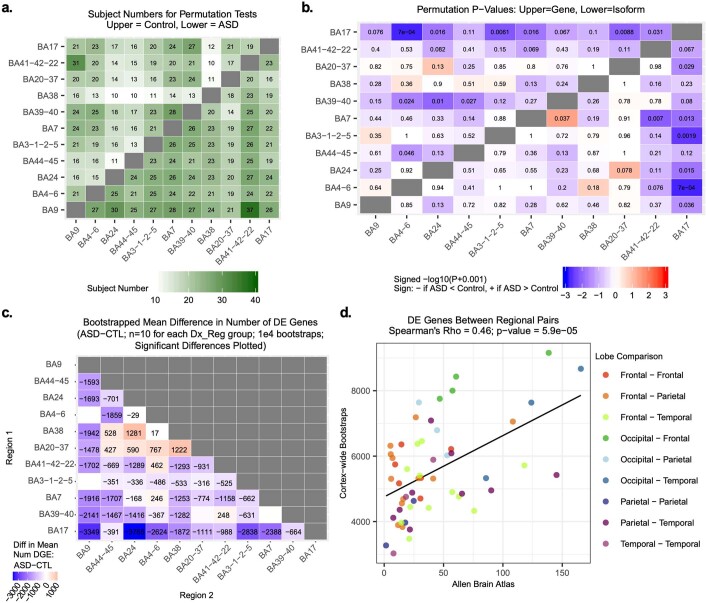
Transcriptomic Regional Identity Attenuation in ASD. a. Sample size for all regional comparisons. b. Nominal permutation derived p-values (two-sided) for all regional comparisons (See Methods). c. Bootstrapped (10,000 bootstraps) mean difference in the number of differentially expressed genes between regions, ASD – CTL (control). Wilcoxon rank-sum test results with FDR < 0.05 are indicated. Negative/blue values = less differentially expressed genes between regions in ASD compared to controls, positive/red = more differentially expressed genes between regions in ASD compared to controls. d. Spearman’s correlation between number of differentially expressed genes between pairs of regions in this study (mean across bootstraps in controls, y-axis) compared to the Allen Brain Atlas (ref. ^13^, mean across matched regions, x-axis; see Methods for matched regions). The Spearman correlation P value (two sided) was calculated using algorithm AS 89.

We first systematically contrasted all unique pairs of 11 cortical regions (55 comparisons in all) using a conservative permutation-based statistical approach to account for differences in sample size across regions (Fig. 2a and Methods). We validated that the observed transcriptomic regional-identity patterns in controls were robust by comparing them with those from the Allen Brain Atlas^13^ (Extended Data Fig. 5, Supplementary Data 4 and Methods). Ten pairs of regions exhibited significantly greater ARI in ASD compared with controls on the basis of permutation, with an additional 41 out of the 55 pairs of regions exhibiting significant attenuation in ASD using another complementary, bootstrap-based approach (Fig. 2b, Extended Data Fig. 5, Supplementary Data 4 and Methods). These results demonstrate that the differences in gene expression that differentiate cortical regions are significantly reduced in ASD, yielding cortical regions that are more molecularly homogeneous (Extended Data Fig. 5).

**Extended Data Fig. 6:**
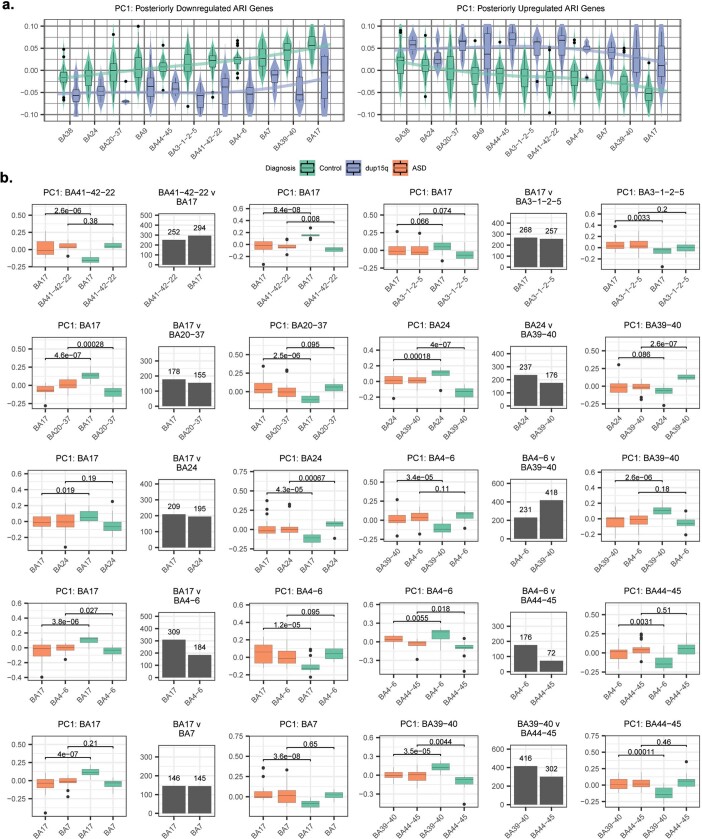
Additional ARI gene dysregulation. a. First principal component (PC1) of ARI genes identified in ASD exhibiting posteriorly downregulated (n = 1,881 genes, left) or upregulated (n = 1,695 genes, right) patterns of expression. Boxplots show median and interquartile range (25^th^/75^th^ percentile; bounds of box) +/− 1.5 times the interquartile range (whiskers) for PC1 of these genes for each regional comparison, summarized across controls and dup15q samples (loess regression line plotted). The unique number of subjects per region is indicated in Fig.1. b. For each significantly attenuated regional comparison, the identified attenuated regional identity (ARI) genes. At center, number of ARI genes with greater neurotypical expression in each pair of regions. On either side of the barplot, the PC1 of the genes with greater neurotypical anterior (left) or posterior (right) expression is plotted across the pair of regions in Controls and ASD. The Wilcoxon signed-rank test (unpaired; two-tailed) p-value is shown. Boxplots show median and interquartile range (25^th^/75^th^ percentile; bounds of box) +/− 1.5 times the interquartile range (whiskers) for PC1 of the genes for each regional comparison, summarized across controls and ASD samples, with the unique number of subjects per group x region comparison indicated in Fig. 1b.

We observed that for nine out of the ten region pairs exhibiting significant attenuation in ASD, one of the posterior regions BA17 or BA39/40 was included (Fig. 2c,d). Notably, BA17 was also one of the regions with the largest case–control differences in gene expression. To determine how gene-expression changes were dispersed across regions in these pairs, we used a conservative filtering process to identify individual representative genes exhibiting ARI (Methods and Supplementary Data 4). Although these genes were widely dysregulated, the more posterior regions, BA17 and BA39/40, exhibited the greatest changes (Fig. 2c,d and Extended Data Fig. 6). ARI genes were also similarly disrupted in the dup15q samples (Extended Data Fig. 6), suggesting that transcriptomic regional-identity attenuation in the cerebral cortex is shared across heterogenous forms of ASD. Together, these results show that in ASD there is a substantial reduction in the typical transcriptional patterns that differentiate cortical regions, with certain posterior regions (BA17 and BA39/40) exhibiting particularly strong patterns of attenuation.

To investigate the biological processes contributing to this broad posterior-predominant ARI gene dysregulation in ASD, we grouped together all of the ARI genes that typically increase in expression along the anterior–posterior axis in controls, but did not in ASD (ARI downregulated: 1,881 genes; Fig. 2c)—or those that decrease in expression along this axis in controls, but not in ASD (ARI upregulated: 1,695 genes; Fig. 2d). Both patterns of up- and downregulation were most prominent posteriorly in ASD, with the largest effects in BA17 followed by parietal cortex (BA39/40). Indeed, ARI genes exhibited significant overlap with differentially expressed genes detected in these regions, much more than in frontal regions (BA17: *P* < 1.8 × 10^−67^; BA39/40: *P* < 1.7 × 10^−16^; BA4/6: *P* = 0.19; Fisher’s exact test). The upregulated set of ARI genes contained several transcription factors (TFs) involved in cortical patterning, including *SOX4*, as well as *SOX11*, which controls postnatal intercortical projection neuron connectivity in mouse via temporal effects on projection neuron maturation^15^. The downregulated set of ARI genes showed broad enrichment for neuronal cell-type-specific markers and RNA-processing pathways, as well as genes with known *ETV4* binding sites (Fig. 2c). Notably, *ETV4*, which is known to have an important role in dendrite development, connectivity and plasticity and its expression—which is normally higher posteriorly (BA17) than anteriorly (BA4/6)—is significantly attenuated, diminishing this gradient in ASD^16^. We further characterize ARI gene dysregulation below via subsequent co-expression network analysis, which further refines the topology and pathways involved.

## Cortex-wide modules harbour ASD risk genes

**Extended Data Fig. 7:**
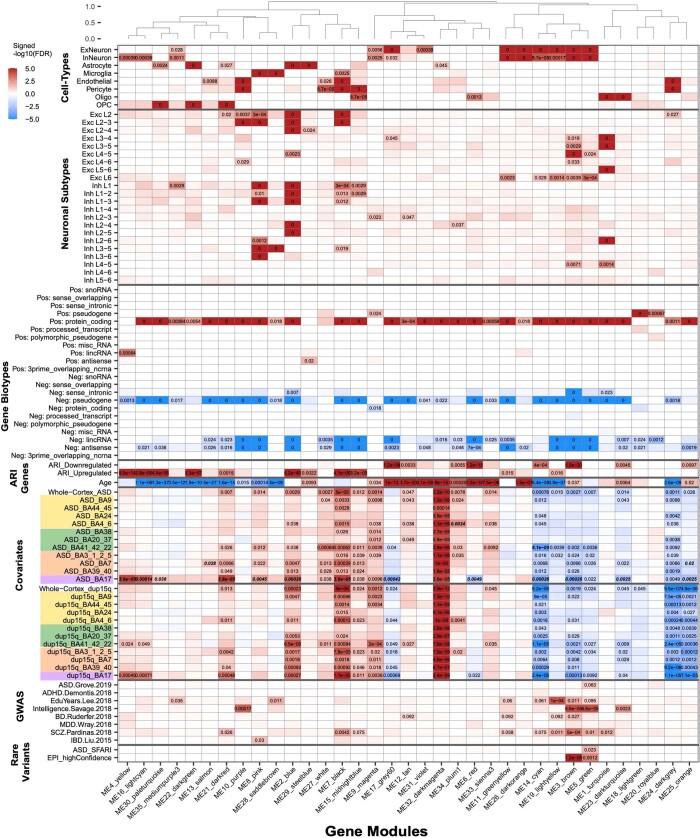
Gene-level Co-Expression Network Analysis Module Associations. Top: average-linkage hierarchical clustering of module eigengene biweight midcorrelations. Significant FDR corrected p-values (two sided) are indicated (FDR < 0.05; for GWAS, FDR < 0.1). For ASD, dup15q, and age covariates, FDR-corrected p-value (two sided) from the linear mixed model testing the association of these covariates with module eigengenes is depicted. For the ASD and dup15q region-specific comparisons, cortical lobule colours are indicated (Fig. 1), and bold-italic FDR-corrected p-values indicate that these regions are affected with significantly greater magnitude than the ASD whole-cortex (Methods). For gene biotypes, both positive and negative enrichment is shown (Fisher’s exact test; Methods). Positive enrichment is shown for cell-type, neuronal subtype (ref. ^23^), ARI gene, GWAS, and rare variant enrichment (Methods).

**Extended Data Fig. 8:**
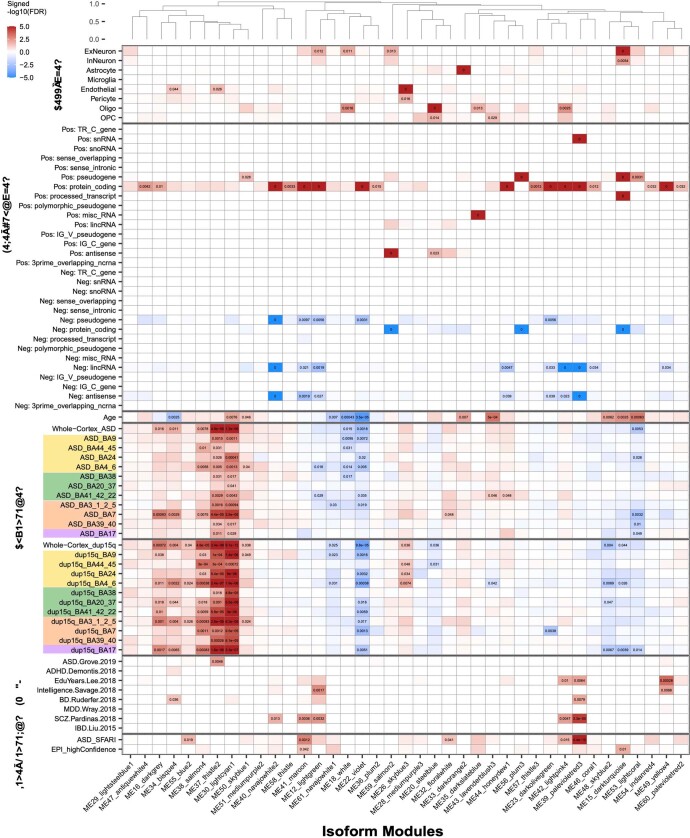
Transcript-level Co-Expression Network Analysis Module Associations. Top: average-linkage hierarchical clustering of module eigengene biweight midcorrelations. Significant FDR corrected p-values (two sided) are indicated (FDR < 0.05; for GWAS, FDR < 0.1). For ASD, dup15q, and age covariates, FDR-corrected p-value from the linear mixed model testing the association of these covariates with module eigengenes is depicted. For the ASD and dup15q region-specific comparisons, cortical lobule colours are indicated (Fig. 1a). For gene biotypes, both positive and negative enrichment is shown (Fisher’s exact test; Methods). Positive enrichment is shown for cell-type, GWAS, and rare variant enrichments (Methods).

We next used weighted gene correlation network analysis^17^ (WGCNA) across all samples to partition genes into clusters with high levels of co-expression, called modules. Modules organize individual transcriptional changes into clusters having shared biological functions or co-transcriptional regulation^18–20^ (Methods). We identified a total of 35 gene co-expression modules that can be summarized by their eigengene, which represents the first principal component of gene expression in the module. Nine modules were downregulated and 15 were upregulated in ASD (Extended Data Fig. 7 and Supplementary Data 5 and 6). We also generated networks using gene transcript-level (isoform) quantifications, which reflect both alternative splicing and alternative promoter usage, identifying 61 transcript modules (Methods). Of these, 39 transcript modules did not overlap with a gene module, among which 5 were downregulated and 9 were upregulated in ASD relative to controls (Extended Data Fig. 8 and Supplementary Data 5 and 6). In total, 38 modules were up- or downregulated in at least one region in ASD. Most of these fell into two broad groups: (1) dysregulated cortex-wide with comparable magnitude across regions (18 modules); or (2) exhibiting variable changes across regions (13 modules). In support of earlier findings in frontal and temporal lobes, dup15q changes were similar to those observed in ASD, but were greater in magnitude (Extended Data Figs. 7 and 8 and Supplementary Data 6).

**Fig. 3:**
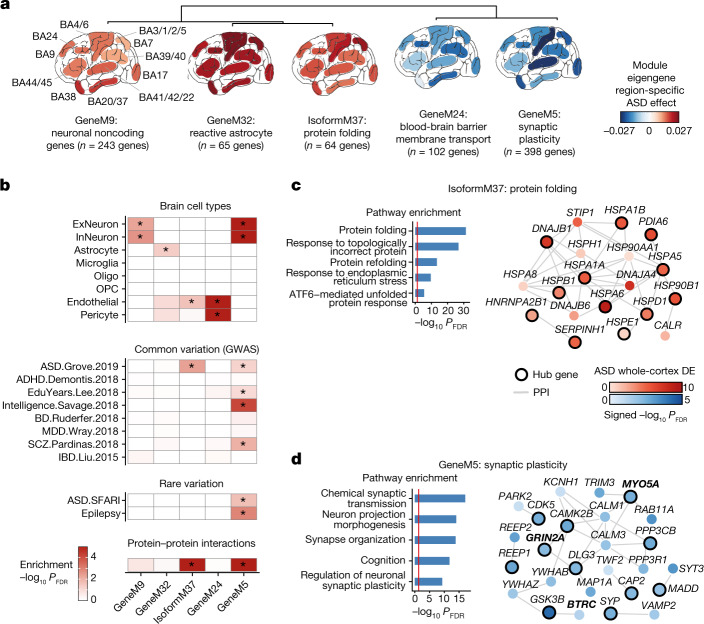
Co-expression network analysis characterizes cortex-wide dysregulation of ASD risk genes. **a**, Hierarchical clustering of the top 5 most dysregulated gene and isoform co-expression module eigengenes (first principal component of the module) with regionally consistent patterns of ASD dysregulation. The module eigengene ASD effect is indicated for each cortical region examined (Methods). *n* indicates the number of genes or isoforms in each module. **b**, −log_10_(FDR) for cell-type, genome-wide association study (GWAS), rare-variant and protein–protein interaction enrichment for the modules depicted in **a**. GWAS references (left margin): ASD.Grove.2019, ref. ^21^;ADHD.Demontis.2018, ref. ^39^; EduYears.Lee.2018, ref. ^40^; Intelligence.Savage.2018, ref. ^41^; BD.Ruderfer.2018, ref. ^42^; MDD.Wray.2018, ref. ^43^; SCZ.Pardinas.2018, ref. ^44^; IBD.Liu.2015, ref. ^45^. *Significant enrichment (FDR < 0.05 for cell-type, rare-variant and protein–protein interaction enrichment, and FDR < 0.1 for GWAS enrichment). **c**,**d**, For ASD GWAS-enriched modules, IsoformM37 (**c**) and GeneM5 (**d**), top gene ontology terms (left) and hub genes (module genes within the top 20 genes with the highest correlation with the module’s eigengene) whose gene products participate in a protein–protein interaction with those of any other module gene are depicted along with their PPI partners (right). Node colour is the signed –log_10_(FDR) of the whole-cortex ASD effect, edges denote direct PPIs, and hub genes are indicated with a black outline. SFARI database^22^ gene symbols are in bold.

The 18 gene or transcript modules exhibiting consistent expression dysregulation in ASD across all regions (linear mixed model, FDR < 0.05; Fig. 3a, Extended Data Figs. 7 and 8 and Supplementary Data 6) include: GeneM9, an upregulated neuronal module with a significant enrichment for non-protein-coding genes; GeneM32, a strongly upregulated module representing reactive astrocytes; and GeneM24, a downregulated module enriched for endothelial and pericyte marker genes involved in blood–brain-barrier functions (Fig. 3b, Extended Data Fig. 7 and Supplementary Data 6). These modules replicate previous findings of neuronal changes, astrocyte reactivity and blood–brain-barrier disruption in ASD^1,4–6^, but extend these findings by demonstrating that these processes are widespread across the cerebral cortex and not restricted to frontotemporal association areas.

Two of the modules demonstrating cortex-wide dysregulation—GeneM5 and IsoformM37—exhibited significant enrichment for ASD-associated common genetic variation^21^ (Fig. 3b–d). GeneM5 is downregulated in ASD, contains many neuronal genes involved in synaptic vesicle function, cytoskeleton and synaptic plasticity, and significantly overlaps with the downregulated synaptic module CTX.M16, previously identified by Parikshak et al.^5^ in frontal and temporal cortex (Fig. 3a,d and Supplementary Data 5 and 6). In addition to common genetic variation, GeneM5 is also significantly enriched for genes containing rare de novo protein disrupting mutations associated with ASD, including the high-confidence risk genes *GRIN2A*, *MYO5A* and *BTRC*^22^ (Supplementary Data 5 and 6 and Methods). GeneM5 is enriched in cortical excitatory and inhibitory neuron cell-type markers^23^ (Extended Data Fig. 7), identifying them as a point of convergence for rare and common genetic risk in ASD. By contrast, IsoformM37 is enriched for ASD common genetic risk variants (but not rare mutations), is upregulated in ASD, and contains genes involved in heat-shock responses and protein folding (Fig. 3a,c and Supplementary Data 6). To our knowledge, this is the first report of an upregulated ASD transcriptomic signature that is associated with known ASD risk variants, and it implicates protein homeostasis as being dysregulated in ASD.

## Regional variation

**Fig. 4:**
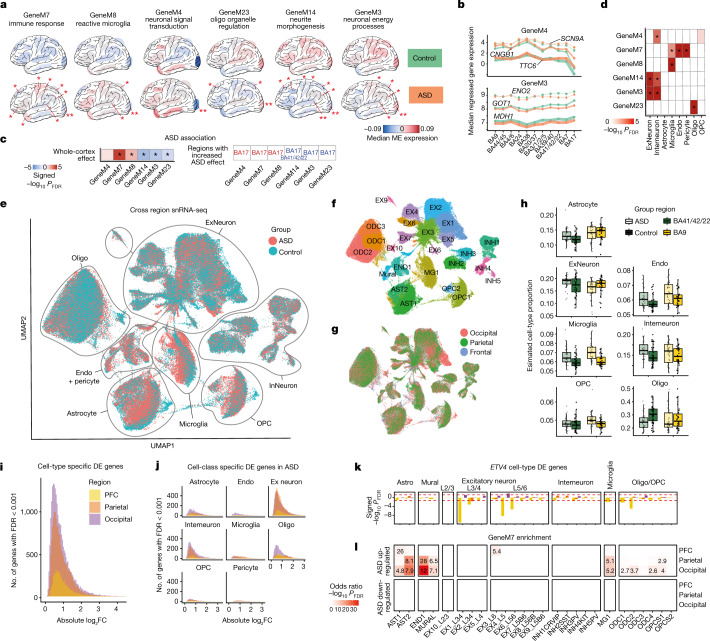
Functional characterization of regionally variable transcriptomic dysregulation in ASD. **a**, The top 6 most dysregulated modules with regionally variable patterns of dysregulation. The median of the module eigengene (ME), stratified by diagnosis, is depicted for each cortical region examined. *Significant region-specific dysregulation in ASD; **regions with a significantly increased magnitude of effect compared to the whole-cortex effect (Methods). **b**, Median regressed gene expression for the top three hub genes for GeneM4 (top) and GeneM3 (bottom). **c**, The whole-cortex ASD effect for modules depicted in **a** (*FDR < 0.05). Bottom, regions with a significantly increased magnitude of effect compared to the whole-cortex effect in **a** are listed. **d**, Cell-type enrichment for regionally variable modules. **e**, Uniform manifold approximation and projection for dimension reduction (UMAP) plots of snRNA-seq data from around 250,000 cells containing matched ASD and neurotypical control samples across frontal, parietal and occipital cortices, coloured by diagnosis. **f**,**g**, UMAP plots coloured by specific cell type (**f**) and cortical region of origin (**g**). **h**, Broad neural cell-type proportions deconvolved from matching bulk methylation array data. No FDR-significant cell proportion shifts were observed in ASD. **i**, Region-specific effect-size changes for cell-type-specific differentially expressed genes (FDR < 0.001), with BA17 again showing the greatest transcriptomic changes. **j**, Differentially expressed genes are shown for broad cell classes. **k**, *ETV4* shows posterior-predominant downregulation across multiple cell types in ASD. **l**, The immune module GeneM7 shows posterior-predominant enrichment among cell-type-specific differentially expressed genes in ASD.

We found 13 modules that exhibited regionally variable patterns of dysregulation in ASD (Fig. 4a–c, Extended Data Fig. 7, Supplementary Data 6 and Methods), all of which also showed anterior–posterior gradients of expression in neurotypical samples. None of these modules, however, were significantly enriched for known ASD genetic risk variants. The most pronounced alterations were observed in BA17, with four modules exhibiting significant associations with ASD that were only detectable in this region. These include GeneM30, an OPC module with hub genes including *SOX4* and *SOX11*. Another, GeneM4, is an inhibitory neuron module containing many genes important for various intracellular signalling and maturation processes, such as *SCN9A* (Fig. 4a–d and Supplementary Data 5 and 6). GeneM4 is significantly enriched with long intergenic non-coding RNAs (lincRNAs) and for previously reported gene modules associated with upregulated pathways related to development^5^ and signalling^1,6^ in ASD, although we observe this effect in BA17 for the first time (Extended Data Fig. 7).

Twelve regionally variable modules showed significant enrichment for genes comprising the ARI signal, indicating that these modules contribute to transcriptomic regional identities that are observed in neurotypical controls, but are attenuated in ASD (Extended Data Fig. 7 and Supplementary Data 6). Six of these modules were more highly expressed in posterior regions relative to anterior regions in neurotypical subjects and were observed to be downregulated in ASD across the cortex (Fig. 4a–d, Extended Data Fig. 7 and Supplementary Data 6). These included GeneM3, a neuronal module enriched for energy generation and neuronal processes that are highly energy dependent, such as vesicle transport and release. Four modules were more highly expressed in anterior regions relative to posterior regions in neurotypical subjects and exhibited cortex-wide upregulation in ASD that attenuated this pattern **(**Fig. 4a–d, Extended Data Fig. 7 and Supplementary Data 6). These include GeneM8, a microglial module containing genes involved in immune signalling and phagocytosis; and GeneM7, an immune response module containing genes such as NF-κB and interferon response pathways. Although neuronal and oligodendrocyte downregulation along with immune and microglia upregulation have been previously reported in ASD^1,4–6^, these findings indicate that this dysregulation is widespread across the cerebral cortex, with increased magnitude in posterior regions, a pattern that is most pronounced in BA17.

We next sought to determine the driver of the observed changes in magnitude of ASD effect across regions. It is well established that BA17 is the most neuronally dense region in the human brain, with a notable expansion in the thickness of layer 3/4 (L3/4), compared with other cortical regions^18^. Similarly, there is an anterior–posterior gradient of increasing neuronal density observed in mice and primates^24–27^. We posited that regional variation in neuronal density or laminar thickness could be contributing to regional differences in the magnitude of the ASD effect. Regional neuronal density across multiple brain regions has not been quantitatively characterized in the human brain, but such gradients have been established across some regions in non-human primates^25,26^. We therefore compared the region-specific ASD effect-size changes in our gene modules to regional neuronal nuclei density measured in primates^25^ for six matched regions across species. We observed a significant correlation between L3/4 thickness, neuronal density and the effect sizes for several modules dysregulated in ASD (Supplementary Data 7).

## Cell-type-specific expression gradients

**Extended Data Fig. 9:**
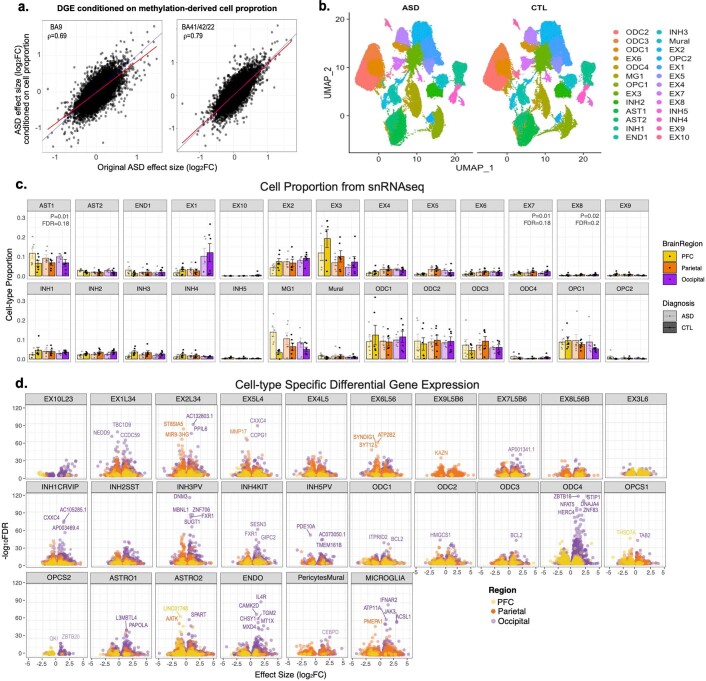
Cell-type Composition Associations, snRNA-seq, and Cell-type Deconvolution. **a**. ASD bulk RNA-seq log_2_FC’s (from BA9, left, and BA41-42-22, right) calculated without (x-axis) and with (y-axis) methylation data derived cell-type proportions as covariates (Methods), compared to the original model used for calculating DE genes (Fig. 1). Spearman correlation is shown. **b**. Split UMAP plots of snRNA-seq from ASD cortex (left) and CTL cortex (right) with cell subtypes coloured. **c**. Cell-type proportion (mean + standard error) for cell clusters from each region derived directly from the snRNA-seq data (n = 12 biologically independent individuals, 6 per group, sampled across 3 brain regions). Region is coloured and cell-types are indicated in each plot. Nominal and FDR-corrected P values from ANOVA (two sided) are shown for trend-level associations. **d**. Volcano plots show within cell-type differential expression across regional comparisons. X-axis is ASD effect size (log_2_FC) and y-axis is the -log_10_(FDR) from Nebula. Genes with |log_2_FC| > 0.2 and FDR < 0.001 are labeled.

These observations motivated us to perform snRNA-seq in a subset of subjects to help evaluate the extent to which changes in gene expression reflect changes in cell proportions in ASD and how distinct neural cell types were contributing to the regional variance in ASD transcriptomic dysregulation identified with bulk RNA-seq (Fig. 4e–g, Extended Data Fig. 9, Supplementary Data 8 and Methods). We sequenced more than 250,000 nuclei from 6 individuals with ASD manifesting strong differential expression signatures and 6 matched control subjects across frontal, parietal and occipital cortices with matching bulk RNA-seq. From these data, we identified 26 distinct cell clusters that represent all expected cortical cell types and regionally specific excitatory cell types in the occipital cortex (Fig. 4f, Supplementary Data 8 and Methods). As expected from the non-human primate data, we observe that superficial excitatory neurons (EX1 L3/4) are increased in proportion by more than 5% in the occipital region in both control and ASD subjects compared with frontal (prefrontal cortex (PFC)) and/or parietal regions (Fig. 4f,g and Supplementary Data 8). These results are consistent with the known increase in thickness of L3/4 in BA17 compared with other cortical regions^28^.

We next performed cell composition analyses using snRNA-seq data to determine whether cell-type proportions were shifted in ASD and could explain the observed underlying bulk RNA-seq signatures. Although there were nominal ASD-associated increases in astrocyte (AST1) and decreases in lower-layer excitatory neuronal (EX7 and EX8) proportions, the changes were of small magnitude and reflect a nominally significant trend (FDR-corrected *P-*values > 0.18; Extended Data Fig. 9 and Supplementary Data 8). To confirm these results in a larger cohort (*n* = 36 per group), we further performed cell-type deconvolution (CTD) using bulk frontal and temporal cortex methylation profiling data with cell-type-specific signatures derived from single-cell methylome analyses in human brain^29^. Methylation CTD analyses detected nominal increases in PFC microglia and decreases in temporal lobe oligodendrocytes in ASD, but no cell proportion shifts survived FDR correction (Fig. 4h and Supplementary Data 7). Finally, to directly evaluate how cell-type proportions may have contributed to our observed ASD effects in the bulk RNA-seq data, we compared ASD log_2_FCs calculated from our original linear model with and without methylation CTD cell-type proportions as covariates (Extended Data Fig. 9, Supplementary Data 7 and Methods). Spearman correlations between ASD log_2_FCs with and without methylation CTD were high (*ρ* > 0.7; Extended Data Fig. 9 and Supplementary Data 7), indicating that cell-type proportions do not significantly affect the ASD-related changes we observed with the bulk RNA-seq data. Although these results may point to a few subtle changes in cortical cell-type proportions in ASD, changes in cell proportions cannot explain the widespread transcriptomic signature observed in bulk RNA-seq.

An alternative explanation is that bulk transcriptomic signatures and modules associated with ASD primarily reflect within-cell-type gene-expression changes that are most detectable in posterior regions. To assess this using the snRNA-seq data, we calculated cell-type-specific differential gene-expression signatures in ASD across frontal, parietal and occipital regions (Methods). Here again, we observed a remarkable regional gradient, with threefold to fourfold more differentially expressed genes observed in occipital and parietal cell types than in PFC (*n* = 23,144, 17,042 and 5,235 genes differentially expressed in total across 26 cell clusters at FDR<0.001, respectively; Fig. 4i, Supplementary Data 8 and Methods). This pattern was consistent following down-sampling to ensure equal cell numbers across comparisons. The majority of differential expression changes came from excitatory neurons across regions, with excitatory neuron classes in the occipital lobe exhibiting the greatest differential expression signal overall, both in terms of the number of differentially expressed genes and effect sizes (Fig. 4i,j and Supplementary Data 8). As an example, we observe that transcription factor *ETV4*—one of the ARI genes in the bulk RNA-seq—shows significant downregulation across multiple distinct occipital cell types, including excitatory neurons and oligodendrocytes (Fig. 4k and Supplementary Data 8). Finally, we find a notable overlap with the bulk RNA-seq co-expression findings, with 90 out of the 96 gene and isoform modules exhibiting significant enrichment among cell-type-specific differentially expressed genes (FDR-corrected *P* < 0.05, Fisher’s exact test; Supplementary Data 8 and Methods). For example, the ASD risk-gene harbouring module GeneM5 was strongly enriched for cell-type-specific differential gene-expression signal across 21 cell classes, particularly including downregulated genes found across multiple oligodendrocyte and excitatory neuron subtypes. Likewise, the ASD GWAS-enriched heat-shock isoform module (IsoformM37) showed significant overlap with upregulated genes across nearly all cell types. These enrichment patterns were also observed for regionally specific modules. For example, the posterior-predominant immune module, GeneM7, is enriched for ASD differential expression signal across all major glial cell types, with a posterior-predominant pattern of enrichment (Fig. 4k).

Thus, by performing multi-region snRNA-seq and CTD, we show that predicted cell-type proportions, as well as cell-type-specific gene-expression profiles are affected across the ASD cerebral cortex. Notably, we see that cell-type-specific transcriptomic dysregulation contributes substantially to the changes observed with bulk RNA-seq, whereas cell-type proportion contributions are subtle (Extended Data Fig. 9 and Supplementary Data 7 and 8). Although cell-type-specific transcriptomic changes and cell-type proportion changes both contribute to ASD gene-expression patterns observed with bulk RNA-seq, our analyses indicate that cell-type-specific gene-expression alterations explain the majority of the observed changes in bulk tissue gene expression.

## Discussion

**Extended Data Fig. 10:**
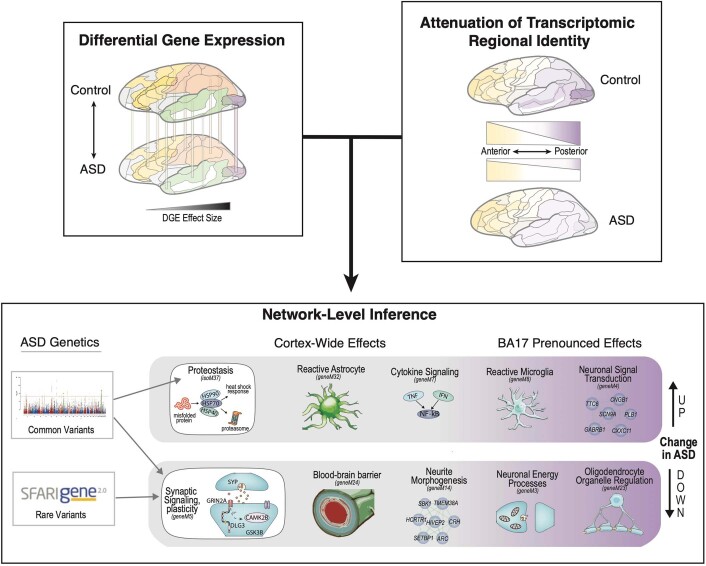
Results Summary. Overview of results from this pan-cortical characterization of the ASD transcriptome. Top left, differential gene expression signatures in ASD, while observed cortex-wide, show greatest effect size changes in posterior regions, especially BA17. Top right, a widespread posterior-predominant attenuation in transcriptomic regional identity is observed in ASD. Bottom, co-expression networks provide an organizing framework for interpretation of cortex-wide and regionally variable effects. A cortex-wide, upregulated co-expression module (isoM37) comprised of genes involved in proteostasis is enriched for common ASD-associated genetic variation. A cortex-wide, downregulated module characterized by genes involved in synaptic signaling and plasticity showed enrichment for common and rare genetic risk variants. Other modules exhibiting cortex-wide or regionally pronounced differential expression patterns in ASD are highlighted.

The findings presented here substantially refine our understanding of ASD molecular pathology beyond the previously established ‘downregulated neuron’ and ‘upregulated glia/immune’ functional categories observed in frontal and temporal lobes^1,4–6,30^. We identify gene and transcript expression changes in ASD that occur across the cerebral cortex, affecting many neural cell types and specific biological processes (Extended Data Fig. 10), extending beyond higher-order association areas to include primary sensory areas, most notably in BA17^1,4–6^. We find that the recently observed gene-expression signatures of upregulated, reactive astrocytes and downregulated blood–brain-barrier membrane transport^1^ are present cortex-wide in ASD. Surprisingly, the most profound gene-expression changes in ASD were observed in the primary visual cortex (BA17). It is interesting to speculate that the substantial changes observed in primary sensory regions may relate to the widespread sensory processing differences in ASD, which are so pervasive that they have been included in the DSM-5 diagnostic criteria^31^.

We find that upregulated immune response and reactive microglia genes, along with downregulated neurite morphogenesis and neuronal energy pathway genes, are not only affected cortex-wide in ASD, but also exhibit a regional gradient that reflects fundamental elements of cortical cytoarchitecture. Notably, the magnitude of regional differential expression changes in ASD parallels the observed patterns of ARI, consistent with them representing manifestations of a common underlying biological process. Given these results, along with our observations of pervasive neuronal dysregulation present throughout the ASD cortex, future work should determine how changes in ASD risk genes affect cortical patterning and connectivity. This is especially salient as ARI in ASD may be a manifestation of an early developmental alteration in cortical arealization, which involves both cell intrinsic factors (for example, genetic or epigenetic regulatory programmes) and responses to extrinsic signals, such as morphogen gradients and thalamic inputs^27^. Given the connection between regional cytoarchitecture, local circuits and long-range brain connectivity^32,33^, parsimony suggests that in addition to developmental patterning contributions^5,20^, the diminution of transcriptomic regional identity reflects changes in local neuronal circuit function, and changes in synaptic homeostasis that are widely propagated^32^. This is supported by the observation that the GeneM5 co-expression module, representing synaptic plasticity genes, is downregulated cortex-wide in ASD. Further, GeneM5 is enriched for genes harbouring both common and rare ASD-associated risk variants, consistent with the downregulation of this module having a causal role in ASD. Although a single, post-mortem snapshot cannot distinguish between the above mechanisms, our results provide a strong rationale for their further experimental investigation, such as with pan-cortical cell-type-specific genomic profiling of the human brain during early development^34^, and developing organoid systems that recapitulate regional identities.

Several technical and biological considerations should guide the interpretation of these results. The samples used were obtained from heterogeneous post-mortem cortical tissue, representing a broad range of subjects across both sexes spanning 2 to 68 years of age. Rigorous methodology was used to account for biological and technical variability, ensuring that the results reported here are conservative and generalizable. To address the issues of cellular resolution and dissection variability across cortical regions, we performed snRNA-seq, further enhancing our understanding of regional variation in ASD transcriptomic dysregulation. However, snRNA-seq experiments typically have fewer unique samples than bulk RNA-seq experiments, and the comparability of snRNA-seq cell-type proportions to true cell-type proportions is currently unclear^35^. It is also challenging to estimate transcripts quantitatively using single-cell RNA-seq approaches, whereas this remains a strength of bulk tissue RNA-seq, especially when coupled with network analysis^36^. Leveraging this, we subsequently identified an upregulated transcript-specific co-expression module enriched with ASD GWAS variants, implicating protein folding dysfunction for the first time as a putative pathway contributing to causal mechanisms of ASD. Notably, upregulated proteostasis is also implicated in Down syndrome^37^, and similar heat-shock machinery has been identified a potential druggable target in tuberous sclerosis complex^38^, indicating that this may be an affected biological process across multiple neurodevelopmental disorders. Employing methods with greater cellular resolution will be necessary for further refinement of the results presented here to specific cortical cell types. As we seek to gain a complete understanding of ASD neural pathology, future approaches that integrate different sources of biological data—including this cortex-wide transcriptomic resource—to determine how ASD risk genes affect the brain will be essential.

## Methods

### Sample acquisition and preparation for RNA-seq

Post-mortem cortical brain samples were acquired from the Harvard Brain Bank as part of the Autism BrainNet project, formerly the Autism Tissue Project (ATP), and the University of Maryland Brain Banks (UMDB). Sample acquisition protocols were followed for each brain bank, and samples were de-identified before acquisition and thus exempt from IRB review. A total of 842 samples from individuals with ASD or dup15q syndrome, and non-psychiatric controls (112 unique subjects) across 11 cortical regions encompassing all major cortical lobes—frontal: BA4/6, BA9, BA44/45, BA24; temporal: BA38, BA41/42/22, BA20/37; parietal: BA3/1/2/5, BA7, BA39/40; and occipital, BA17—were acquired. These included 253 samples previously published collected by Parikshak et al. (2016)^5^ from BA9 and BA41/42/22 and/or Gandal et al. (2018)^1,5^ from BA9, BA4/6 and BA41/42/22. An ASD diagnosis was confirmed by the Autism Diagnostic Interview–Revised (ADIR) in 30 of the subjects. In the remaining 19 subjects, diagnosis was supported by clinical history. Additional samples with ‘NCTL’ diagnoses (samples with Angelman’s syndrome, certain CNVs, or epilepsy—but not ASD) were obtained and sequenced, but these samples were ultimately not utilized for the analyses presented here (but they are included in the raw data files accompanying this work). Frozen brain samples were stored at −80 °C. To extract RNA from these samples, approximately 50–100 mg of tissue was dissected from the cortical regions of interest on dry ice in a dehydrated dissection chamber to reduce degradation effects from sample thawing and/or humidity. RNA was then isolated using the miRNeasy kit with no modifications (Qiagen). For each RNA sample, RNA quality was quantified using the RNA integrity number (RIN) on an Agilent Bioanalyzer.

### RNA-seq and RNA data processing

Initial sequencing of BA9 and BA41/42/22 samples was performed in three batches as described^5^. For those batches, starting with total RNA, ribosomal RNA (rRNA) was depleted (RiboZero Gold, Illumina) and libraries were prepared using the TruSeq v2 kit (Illumina) to construct unstranded libraries. The remaining samples (additional brain regions and technical replicates from BA9 and BA41/42/22) were sequenced across three new batches, with strand-specific libraries prepared using the TruSeq Stranded Total RNA sample prep kit with rRNA depletion (RiboZero Gold, Illumina). All libraries were randomly pooled to multiplex 24 samples per lane using Illumina TruSeq barcodes. Each lane was sequenced 5 times on an Illumina HiSeq 2500 or 4000 instrument using high-output mode with standard chemistry and protocols for 50, 69 or 100 bp paired-end reads (read length varied by batch) to achieve a target depth of 70 million reads (see Extended Data Fig. 2a).

After sequencing, the resulting sample FASTQ files from all batches (including the Parikshak et al.^5^ samples) were subjected to the same processing pipeline. First, FASTQ files were assessed with FastQC^46^ (v0.11.2) to verify that quality was sufficient for further processing. FASTQ files were then aligned to the human reference genome (GRCh37^47^ Ensembl v75) with STAR^48^ (v2.5.2b). Picard tools (v2.5.0) was used with the resulting BAM files to collect various read quality measures, in addition to the quality measures collected by STAR. verifyBAMID^49^ was also used with these BAM files along with known sample genotypes from Parikshak et al.^5^ to validate that sample identity was correct for all BAM files. Additionally, the expression of *XIST* (a female-specific gene) was assessed to contribute to sample identity verification. Finally, RSEM^50^ (v1.3.0) was used for quantification (Gencode^51^ release 25lift37) to obtain expected read counts at the gene and transcript levels.

Expected gene and transcript read counts were then subjected to several processing steps in preparation for downstream analysis, mainly using R^52^. First, counts per million (CPM) were obtained from counts for gene and transcript filtering purposes. Genes and transcripts were filtered such that genes and transcripts with a CPM > 0.1 in at least 30% of samples were retained. Genes and transcripts were also removed which had an effective length (measured by RSEM) of less than 15 bp. Transcripts were additionally filtered such that all transcripts corresponded with genes in the gene-level analysis. The counts for the remaining genes (24,836) and transcripts (99,819) passing these filters were normalized using the limma-trend approach in the limma^53^ R package. Briefly, the limma-trend approach obtains normalized expression data through taking the log_2_(CPM) of read counts with an adjustment for sample read depth variance. An offset value calculated with CQN^54^ accounting for GC content bias and gene/transcript effective length bias in read quantification was also incorporated during the normalization process. With this normalized expression data, sample outliers were identified in each sequencing batch by cortical lobe (frontal, parietal, temporal, and occipital) group that had both (1) an absolute *z*-score greater than 3 for any of the top 10 expression principal components and (2) a sample connectivity score less than −2. Sample connectivity was calculated using the fundamentalNetworkConcepts function in the WGCNA^17^ R package, with the signed adjacency matrix (soft power of 2) of the sample biweight midcorrelation as input. This process identified 34 outliers, resulting in a final total of 808 samples (341 control, 384 ASD and 83 dup15q), which were carried forward for analysis.

### Evaluating ASD differentially expressed genes and transcripts cortex-wide

Linear models for all subsequent analyses are described in the Supplementary Methods. The limma^53^ R package was used to identify differentially expressed genes and transcripts in ASD both within specific regions and cortex-wide, controlling for known biological and sequencing related technical covariates. Biological covariates included: diagnosis, region, sequencing batch, sex, ancestry, age and age squared. Technical covariates are listed in the Supplementary Methods. The limma::duplicateCorrelation function was used to account for the non-independence of samples derived from the same subject across multiple brain regions. For both region-specific and whole-cortex effects, genes or transcripts with an FDR-corrected *P*- value of <0.05 were considered significantly dysregulated. dup15q region-specific and whole-cortex dysregulation was also established in this manner. The fixed effects of sex, age and age squared were also acquired using the full gene and transcript models (Supplementary Data 3).

To compare the magnitude of the ASD transcriptomic signature across regions, we sought to compute the slope of the linear regression of ASD effect-size (log_2_FC) changes between whole-cortex and regional comparisons **(**Fig. 1e). However, the linear regression slope is dependent on the (arbitrary) ordering of the response (*Y*) and predictor (*X*) variables, both of which are estimated with error, and we found that in practice the slope can change considerably according to this order. To circumvent this issue, we used total least square regression (also known as orthogonal regression), which provides an estimate of slope that is invariant to the choice of predictor and response variables, as we have previously published^6^ (see Supplementary Methods).

### Transcriptomic regional-identity analysis

To identify differentially expressed genes and transcripts between all 55 pairs of cortical regions with our permutation-based approach, a regressed gene-expression dataset containing only the random effect of subject and the fixed effects of diagnosis and region (along with the model residual) was used. This regressed dataset was created with the ‘lmerTest’^55^ package in R through subtracting the effects of technical covariates and all biological covariates other than subject, diagnosis, and region from each gene, leaving only the random intercept, these three remaining biological covariate effects, and the residual. Significant attenuation of differentially expressed genes between each pair of regions (a reduction in transcriptomic regional-identity differences) in ASD was established through the following process. (1) ASD and control subjects containing each region in the regional pair were extracted for use in the analysis. (2) Separately in ASD and control subjects, the number of differentially expressed genes between regions was calculated using the paired Wilcoxon signed-rank test. Genes with an FDR-corrected *P*-value < 0.05 were considered differentially expressed. (3) The difference in the number of differentially expressed genes between regions for ASD vs control subjects was calculated (the ‘true’ difference). (4) A permuted distribution of the difference in differentially expressed genes between regions for ASD vs control subjects was generated to test the ‘true’ difference. Each permutation (10,000 in total) randomly assigned ‘ASD’ and ‘control’ status to subjects but kept the number of ASD and control subjects consistent with the true number of ASD and control subjects. (5) A two-tailed *P*-value was obtained from testing the ‘true’ difference against the permuted distribution. If the regional comparison *P*-value < 0.05, with the number of differentially expressed genes between regions in ASD less than that in controls, then the regional comparison was considered significantly attenuated in ASD. Otherwise, the regional comparison was considered over-patterned in ASD. This procedure was repeated with transcript-level regressed gene-expression data (similarly, only containing the random effect of subject and the fixed effects of diagnosis and region, along with the model residual) to identify altered transcriptomic identities in ASD at the transcript level.

The previously described permutation approach was designed to identify differences in transcriptomic regional identity in ASD. Importantly, this method is not appropriate for assessing variance in expected numbers of differentially expressed genes between regions across regional pairs and diagnoses, since the number of ASD and control subjects varied across regional pairs. To examine this, we also implemented a bootstrap-based approach. For each regional comparison we subset to ten pairs of ASD and control subjects (ten was selected since every regional comparison had at least this many subjects). When subsetting, subjects were removed such that the remaining subjects were closest in age to the median age of the available samples for that regional comparison. A bootstrap approach was then used to calculate the number of differentially expressed genes (Wilcoxon test for all genes, FDR < 0.05) between regions separately in control and ASD subjects through sampling subjects with replacement. After 10,000 bootstraps, control and ASD distributions were compared with Wilcoxon tests to determine if there was significant attenuation of regional identities (an FDR < 0.05 for the 55 regional pair Wilcoxon tests comparing the differentially expressed gene distributions from the bootstraps). The same regressed expression dataset used for the permutation approach was utilized for this bootstrap analysis. Any regional comparison in which the number of differentially expressed genes between regions was less in ASD than in control subjects, and the FDR-corrected *P*-value was less than 0.05, was considered attenuated in ASD.

To validate our bootstrapped estimates for the number of differentially expressed genes between pairs of regions in controls, we compared these estimates to those of the Allen Brain Atlas^13^, which is the best publicly available work for comparison. Allen Brain Atlas regions were matched to Brodmann regions (Supplementary Data 4) and matching regional pairs were extracted for comparison with this work. When the Allen Brain Atlas had two or more regional pairs matching one regional pair in this work, the mean was taken across the Allen Brain Atlas regional pairs. A *P*-value for the association of the number of differentially expressed genes between regions in Controls obtained in this work compared to the Allen Brain Atlas was calculated from a linear model (cortex-wide bootstrap mean ~ Allen Brain Atlas mean).

We applied a stringent filtering process to identify high-confidence ARI genes from each significantly attenuated regional comparison identified with the permutation procedure described above. First, for each of the attenuated regional comparisons, we extracted the genes which were identified as differentially expressed between regions in subjects labelled as controls in each of the 10,000 permutations. Then, we calculated how many times each of the genes truly differentially expressed between pairs of regions in the control subjects were present in their respective permuted groups (ranging from a possible 0 to 10,000 occurrences). Those ‘true’ differentially expressed genes which were present in less than 95% of their respective permutations were retained as ARI genes for each attenuated regional comparison. For each set of ARI genes (ten total), each gene was matched to the region in which it had higher expression in control subjects. The paired Wilcoxon signed-rank *P*-values identified for these genes in controls (those subjects used for the permutation analysis) were also extracted and are shared in Supplementary Data 4.

ARI gene groups (ARI downregulated genes, those highly expressed in BA17 and BA39/40 relative to other regions in controls; ARI upregulated genes, those expressed at low level in BA17 and BA39/40 relative to other regions in controls) were created through taking the union (without duplicates) across all ten identified ASD-attenuated regional comparisons, and sorting genes into the two groups based on gene-expression profiles across regions. The details of this process are described in the Supplementary Methods, along with functional annotation procedures.

### Network-based functional characterization

Standard workflows using WGCNA^17^ were followed as previously described in Parikshak et al.^5^ and Gandal et al.^1^ (with minor modifications) to identify gene and transcript co-expression modules. Details regarding network formation, module identification, and module functional characterization are described in the Supplementary Methods.

### snRNA-seq

Frozen brain samples were placed on dry ice in a dehydrated dissection chamber to reduce degradation effects from sample thawing and/or humidity. Approximately 50 mg of cortex was sectioned, ensuring specific grey matter–white matter boundary. The tissue section was homogenized in RNase-free conditions with a light detergent briefly on ice using a dounce homogenizer, filtered through a 40-μM filter and centrifuged at 1,000*g* for 8 min at 4 °C. The pelleted nuclei were then filtered through a two-part micro gradient (30%/50%) for 20 min at 4 °C. Clean nuclei were pelleted away from debris. The nuclei were washed two more times with PBS/1%BSA/RNase and spun down at 500*g* for 5 min. Cells were inspected for quality (shape, colour and membrane integrity) and counted on a Countess II instrument. They were then loaded onto the 10X Genomics platform to isolate single nuclei and generate libraries for RNA sequencing on the NovaS4 or NovaS2 Illumina machines.

After sequencing, Cell Ranger software (10X Genomics) was used to prepare fastq file and reads were aligned to the human GRCh38 pre-mRNA genome to generate gene by cell matrices for each library. Pegasus (v.1.4.0) was used to stringently filter cells, remove doublets, integrate and batch-correct all libraries together. Cells were removed if they expressed more than 6,000 or less than 750 genes, or had more than 10% mitochondrially mapped reads. Utilizing 65 PCs, Harmony (as part of the Pegasus suite) was used to integrate and batch-correct libraries, Louvain clustering was performed to cluster the cells and visualize resulting clusters with UMAP^56^. Cell types were annotated based on expression of known marker genes visualized via UMAP and by performing unbiased gene marker analysis (Supplementary Data 8). Canonical genes were selected based on mouse and human studies as well as published reference atlas enriched genes^23,57^.

For cell composition analyses of snRNA-seq data, cell fractions were calculated for each sample and then underwent centred-log ratio (clr) transformation, which accounts for compositional data, and values are interpreted relative to the geometric mean^58^. A repeated-measures ANOVA was used to assess group-level significance, controlling for fixed effects of region, sex, age, library size, nGene and percent_mito, with a random effect for subject. Differential expression was assessed using a negative binomial mixed model as implemented in the NEBULA R package (v1.2.0)^59^. We used a model matrix ~ Diagnosis + Age + Sex + nGene + percent_mito, where quantitative data are scaled. Filtered raw counts are provided as input and NEBULA-LN method is used (default values are used for the other options). Final differentially regulated genes are determined by Benjamini–Hochberg corrected *P*-values.

### Methylation-based CTD analyses

CTD was calculated using bulk methylation array data with matched samples^60^ in BA9 and BA41/42/22 (Supplementary Data 7), we performed reference-based deconvolution for seven brain cell types (excitatory neurons, inhibitory neurons, astrocytes, microglia, endothelial cells, oligodendrocytes and oligodendrocyte precursor cells) using single-cell methylome reference data^29^. We chose to emphasize these results in this manuscript as the zero-to-one scaling and the constant number of potentially methylated sites within each cell makes it more amenable to deconvolution than transcriptomic data. We used our methylation CTD cell-type proportions to test if cell-type proportion could explain our observed ASD differential expression effects in the bulk RNA-seq data. We addressed this through adding cell-type proportions as covariates to our linear model for differentially expressed gene analysis. We tried two different models: one including our broad cell-type proportions, and the other including the top two principal components of the cell-type proportions (compositional PCs; Supplementary Data 7). Neither of these models had any substantial effect on the ASD log_2_FC derived from the differentially expressed gene analysis (Extended Data Fig. 9 and Supplementary Data 7). Please see the Supplementary Note for a detailed explanation of these analysis.

### Reporting summary

Further information on research design is available in the Nature Research Reporting Summary linked to this article.

## Online content

Any methods, additional references, Nature Research reporting summaries, source data, extended data, supplementary information, acknowledgements, peer review information; details of author contributions and competing interests; and statements of data and code availability are available at 10.1038/s41586-022-05377-7.

## Supplementary information

Supplementary Information This file contains Supplementary Methods and references.

Reporting Summary

Supplementary Data 1 Available metadata and sequencing quality metrics for all samples from the processed dataset (outliers which were removed are not included).

Supplementary Data 2 Overlap between whole brain and regional differential gene/isoform expression results; comparison with results from Perturb-Seq (ref. 72); Spearman’s correlation of gene-expression PCs with ADI-R scores.

Supplementary Data 3 Summary statistics for whole cortex and region-specific differential gene-expression (DGE) and differential isoform expression (DIE) results from ASD vs control and dup15q vs control comparisons.

Supplementary Data 4 Summary statistics characterizing ARI results in ASD vs control samples. Results from permutation-based and bootstrap analyses, comparison with Allen Brain Atlas, and ARI gene enrichment analyses.

Supplementary Data 5 Results from gene and isoform-level WGCNA. WGCNA module assignment and module membership (kME values), along with gene annotation, for all genes assessed.

Supplementary Data 6 Functional characterization of all gene and isoform modules, based on enrichments for neural cell-types, gene ontologies, gene biotypes, ARI genes, GWAS heritability, and rare variant signal.

Supplementary Data 7 Comparison of regional ASD differential expression signatures with measures of neuronal density and L4 thickness. Summary results from bulk brain methylation data based cell-type CTD in ASD cases and controls.

Supplementary Data 8 Meta-data and summary results from snRNA-seq profiling of ASD and control subjects across 3 brain regions (PFC, Parietal, Occipital cortex). Cluster definitions, UMAPs, and summary statistics from cell-type-specific differential expression analyses.

## Data Availability

The source data (bulk and snRNA-seq) generated in this manuscript are available via the PsychENCODE Knowledge Portal (https://psychencode.synapse.org/) at 10.7303/syn34637740.2. The PsychENCODE Knowledge Portal is a platform for accessing data, analyses and tools generated through grants funded by the National Institute of Mental Health (NIMH) PsychENCODE program. Data are available for general research use according to the following requirements for data access and data attribution: https://psychencode.synapse.org/DataAccess. Single-cell and bulk RNA-seq data from the Allen Brain Atlas were downloaded from http://portal.brain-map.org/. Single-cell methylation data from Luo et al. are available from the Gene Expression Omnibus under accession GSE140493.

## Code availability

The code for the RNA-seq analyses that support the findings of this study are available in a publicly accessible GitHub repository at https://github.com/dhglab/Broad-transcriptomic-dysregulation-across-the-cerebral-cortex-in-ASD.
