# Supplementary material for: Broad transcriptomic dysregulation occurs across the cerebral cortex in ASD
Source: Nature. 2022 Nov 2;611(7936):532–9. doi: 10.1038/s41586-022-05377-7 (PMC9668748; doi:10.1038/s41586-022-05377-7)
Supplement: Supplementary file 1 — This file contains Supplementary Methods and references. [file 41586_2022_5377_MOESM1_ESM.pdf]

nature portfolio

https://doi.org/10.1038/s41586-022-05377-7

Supplementary information

Broad transcriptomic dysregulation occurs across the cerebral cortex in ASD

In the format provided by the
authors and unedited

Supplementary Information

Supplementary Methods

Linear model design

To select the appropriate technical covariates to use in downstream linear mixed-effects models, we employed the stepwise regression technique as implemented in the earth[61] package in R. This package applies the Multivariate Adaptive Regression Splines (MARS) technique to build adaptive regression models with cross-validation. The covariates assessed were subject, region, brain bank, diagnosis, sex, age, PMI, sequencing batch, ancestry genotype, and RIN, as well as STAR and PicardTools RNA-seq quality measures (all listed in **Supplementary Data 1**). For 4 subjects with no recorded PMI, the average of the rest of the subjects' PMI was used. Before input into the EARTH algorithm, STAR and PicardTools quality metrics were filtered such that collinearity with any other biological or technical covariate was eliminated (only one covariate was kept for every identified collinear pair, with collinearity defined as an adjusted $R^2 > 0.95$ between the two covariates). All continuous covariates were centered and scaled for input into the EARTH algorithm and for remaining analyses. A cross-validated approach was used to run EARTH: it was run 10 times with 90% of samples, and then the resulting linear model was tested with the remaining 10% of samples. The median R^2 across all genes/isoforms was used to assess the performance of each cross-validated EARTH model. Using this metric, the following covariates from the highest performing EARTH model were selected for the gene and isoform linear mixed models used in subsequent analyses:

Gene Model: subject, diagnosis, region, sequencing batch, sex, ancestry, age, age^2, PMI, RIN, picard_gcbias.AT_DROPOUT, star.deletion_length, picard_rnaseq.PCT_INTERGENIC_BASES, picard_insert.MEDIAN_INSERT_SIZE, picard_alignment.PCT_CHIMERAS, picard_alignment.PCT_PF_READS_ALIGNED, star.multimapped_percent, picard_rnaseq.MEDIAN_5PRIME_BIAS, star.unmapped_other_percent, picard_rnaseq.PCT_USABLE_BASES, picard_alignment.PCT_CHIMERAS^2, star.uniquely_mapped_percent^2.

Isoform Model: subject, diagnosis, region, sequencing batch, sex, ancestry, age, age^2, PMI, RIN, picard_rnaseq.PCT_MRNA_BASES, picard_gcbias.AT_DROPOUT, picard_rnaseq.PCT_UTR_BASES, star.multimapped_toomany_percent, picard_rnaseq.MEDIAN_CV_COVERAGE, picard_insert.MEDIAN_INSERT_SIZE, picard_rnaseq.PCT_INTERGENIC_BASES, picard_rnaseq.PF_BASES.

For both models, 'subject' was input as a random effects term (specifically, a random intercept), and diagnosis and region were combined to create one 'diagnosis x region' term (e.g., ASD_BA17, ASD_BA9, Control_BA17, Control_BA9, etc.). This was done to facilitate region-specific contrasts in downstream analyses. The rest of the covariates were input as fixed effects into the linear mixed models. The variancePartition[62] R package was used to visualize the percent of variance explained by each model covariate across all genes/isoforms. The whole cortex DGE effect was then modeled using contrasts, as follows:

```
datMeta$DxReg = paste(datMeta$Group, datMeta$Region, sep="_")
design = model.matrix(~ 0 + DxReg + [...], datMeta) #including covariates as above
corfit <- duplicateCorrelation(datExpr,design,block=datMeta_model$Subject) #random effect for subject
fit <- lmFit(datExpr, design, block=datMeta_model$Subject,correlation=corfit$consensus)

this_contrast_asd = makeContrasts(contrast=(DxRegASD_BA17 + DxRegASD_BA20_37 + DxRegASD_BA24 + DxRegASD_BA3_1_2_5 +
                                            DxRegASD_BA38 + DxRegASD_BA39_40 + DxRegASD_BA4_6 + DxRegASD_BA41_42_22 +
                                            DxRegASD_BA44_45 + DxRegASD_BA7 + DxRegASD_BA9 - DxRegCTL_BA17 -
                                            DxRegCTL_BA20_37 - DxRegCTL_BA24 - DxRegCTL_BA3_1_2_5 - DxRegCTL_BA38 -
                                            DxRegCTL_BA39_40 - DxRegCTL_BA4_6 - DxRegCTL_BA41_42_22 - DxRegCTL_BA44_45 -
                                            DxRegCTL_BA7 - DxRegCTL_BA9)/11, levels=design)

fit_asd = contrasts.fit(fit, this_contrast_asd)
fit2_asd= eBayes(fit_asd,trend = T, robust = T)
```

```
sumstats_ASD_DGE_wholecortex = topTable(fit2_asd, coef=1, number=Inf, sort.by = 'none')
```

Comparing region-specific ASD effects to whole cortex ASD effects

To compare region-specific ASD gene dysregulation effect sizes to the whole cortex ASD effect, we used total least squares regression (also called orthogonal regression) to calculate the slope comparing the whole cortex ASD $\log_2$ Fold Change (FC/effect) to the region-specific ASD $\log_2$ FC for the 4,223 genes identified as DE in ASD across the whole cortex. This was implemented using principal component analysis, as follows::

```
pcreg = function(ds1, ds2) {
  #Total least squares (orthogonal regression) implemented using principal component analysis
  r = prcomp(~ds1+ds2)
  slope <- r$rotation[2,1] / r$rotation[1,1]
  intercept <- r$center[2] - slope*r$center[1]
  rho = cor(ds1,ds2,method="spearman")
  return(list(slope,intercept, rho))
}
```

We then generated a bootstrapped distribution (1,000 bootstraps) for each of the 11 region-specific slopes (sampling with replacement from the region of interest for each ‘diagnosis x region’ group) to calculate a 95% confidence interval for these slopes. Sample size was kept consistent for each bootstrap with the number of samples from each ‘diagnosis x region’ group.

Quality Control Checks

We performed additional analysis to determine if ADI-R scores correlate with the magnitude of ASD-related gene expression change within each region. Specifically, we calculated the Spearman correlation for available ADI-R scores with the first and second principal component of differentially expressed genes, for each region independently. We calculated the principal components with a regressed dataset that only contained the effects of biological covariates and the residual (see **Methods**, this was the same dataset used for gene network generation). We note that we have relatively few unique subjects with ADI-R scores available (32 for ADI-R A, C, and D; 16 for ADI-R-B-NV, and 21 for ADI-R-B-V). As we show in **Ext Data Fig. 3b**, generally small negative and positive correlations are both present, with no correlation greater than 0.39 (observed with PC2 of BA9 and the ADI-R B Nonverbal score) and no correlation less than -0.68 (observed with PC1 of BA17 and the ADI-R B Verbal score). Data is in **Supplementary Data 2**.

We also evaluated how some samples with high PMI=96 but that were not removed as outliers effected our conclusions. To do this, we re-ran our DE gene analysis and calculated ASD $\log_2$FC and FDR corrected p-values without the PMI=96 samples for the Whole Cortex, BA9, and BA41-42-22 (only two samples are from this subject, one in region BA9 and the other in region BA41-42-22). These results are included in **Supplementary Data 2**. We found that the number of ASD DE genes are not meaningfully impacted by removing the PMI=96 samples. The biggest change in DE genes was, for our downregulated genes found in the whole cortex with all samples, 38 out of 2279 genes were lost when removing the PMI=96 samples (1.7%). We also evaluated the Spearman and Pearson correlations between ASD $\log_2$FC calculated with and without the PMI=96 samples, and all correlations were equal to 1.

ARI gene group formation and functional annotation

To evaluate the ARI genes more broadly across the anterior-posterior axis of the cortex, instead of only in the regional pairs in which they were identified, the ARI genes from regional pairs containing either BA17 or BA39-40 were assembled into two groups: the union (without duplicates) of ARI genes with higher control expression in BA39-40 and BA17 relative to other regions (posteriorly ASD-downregulated ARI genes), or the union (without

duplicates) of ARI genes with higher control expression in the remaining cortical regions relative to BA39-40 and BA17 (posteriorly ASD-upregulated ARI genes). Genes which were sorted into both groups (eg. highest expression in BA39-40 v. BA44-45 in one regional comparison, and highest expression in BA7 v. BA17 in another) were removed. Additionally, for each remaining ARI gene, the median Control gene expression in BA17 and BA39-40 (from the regressed gene expression dataset used for the permutation analysis, using all Control samples) was compared to the median across all remaining regions. Only ARI genes with higher median expression in their respective group (eg. higher median expression in BA17 and BA39-40 in the posteriorly ASD-downregulated ARI gene group) were retained. For each gene in each of the two groups, the linear contrast comparing BA17 and BA39-40 gene expression to all other cortical regions was assessed in controls with the same linear model workflow and normalized, outlier-removed gene expression dataset used to identify DE genes and isoforms described before. The beta values and p-values from this analysis are shared in **Supplementary Data 4** and for the top attenuated transcription factors (TFs) in **Figure 2c-d**.

To functionally characterize the ARI gene groups, we performed cell-type and gene ontology enrichment, identified transcription factors present, and calculated transcription factor binding site enrichment. Cell-type enrichment was conducted using the Expression Weighted Cell Type Enrichment (EWCE) method,[63] with a single-cell RNA-seq reference as defined by the Lake et al. Nat Biotechnol 2018 [64] (frontal and visual cortex samples combined). We used a broadly defined set of cell-types, corresponding to the annotation 'Level 1' nomenclature in the EWCE package, comprised of: excitatory neurons, interneurons, astrocytes, microglia, oligodendrocytes, oligodendrocyte precursor cells (OPCs), endothelial cells, and pericytes. To obtain cell-type specificity scores, first genes were filtered such that the gene needed to have a mean UMI of 0.005 across all cells. Then, gene UMI averages were taken across all above cell-types, and these averages were used to generate the cell-type specificity scores utilized by EWCE to calculate cell-type enrichment in the ARI gene groups. 100,000 bootstraps were generated to determine the significance of cell-type enrichment with EWCE.

gProfileR[65] was used for gene ontology enrichment, with FDR-adjustment for p-values, strong hierarchical filtering (`hier_filtering = "strong"`), and a required overlap size (`min_isect_size`) of 10 genes. For the ARI downregulated gene group, a max set size of 2500 was enforced, whereas no max set size was enforced for the ARI upregulated gene group. Only 'BP' (biological process) terms were included in **Figure 2** and **Supplementary Data 4**. Transcription factor binding site enrichment was also conducted with gProfileR, with a Bonferroni-adjustment for p-values and strong hierarchical filtering. To identify transcription factors within the ARI gene groups, AmiGo[66] was used to acquire all genes in GO:0003700 (DNA-binding transcription factor activity) in the Homo sapiens organism (Gene Ontology Consortium,[67,68] accessed May 7, 2020).

WGCNA network formation and module identification

Weighted Gene Co-Expression Network Analysis (WGCNA)[17] was conducted to sort observed gene and isoform expression dysregulation into empirically-informed modules which could provide precise functional insight into affected neural cell-types and biological processes. Regressed gene and isoform expression datasets containing only the random effect of subject, the fixed biological effects (diagnosis, region, age, age^2, sex, and ancestry), and the model residual were used for WGCNA signed network generation. This regressed dataset was created with the 'lmerTest'[55] package in R through subtracting the effects of technical covariates from each gene, leaving only the random intercept, biological covariate effects, and the residual. Signed networks preserve gene expression directions of effect (i.e. genes with increased expression in ASD mapped to modules with increased module eigengene values in ASD).

A soft-threshold power of 6 was chosen for gene network generation, whereas a power of 10 was selected for isoform network generation. These values were selected to optimize induced scale-free topology in the gene and isoform networks ($R^2 > 0.8$). For the gene-level WGCNA, a robust version of WGCNA (rWGCNA)[20] was

implemented to mitigate the influence of potential sample outliers in network formation. Subjects within each diagnosis group were randomly selected (with replacement) for inclusion in the adjacency matrix (formulated using the bi-midweight correlation of genes) and subsequent TOM matrix generation, 100 times. These TOMs were merged into one consensus TOM through first using a quantile scale of 0.95 to calibrate each TOM, and then taking the median across all TOMs to create the consensus TOM. To identify modules from the consensus TOM, the 'cutTreeHybrid' function was used with average linkage hierarchical clustering of the consensus TOM, a deep split of 4, cut height of 0.9999, a negative PAMstage, and minimum module size of 50. Modules within a cut height of 0.1 were merged.

Since rWGCNA could not be implemented for the isoform expression data due to memory allocation limitations, the 'blockwiseModules' function was used with 4 blocks (26,000 or less isoforms per block) to generate the isoform network and identify modules. The same module identification parameters (except for the soft power threshold) used for the gene network were also used for the isoform network. To test the robustness of the isoform network, a permutation approach was utilized.[11,20] For each module, this method tests if the mean connectivity within the module (also defined as the module's density, or the average intramodular topological overlap) is significantly different from that of modules of equivalent size randomly selected from the same network (n=5,000 permutations). One-tailed p-values were calculated through comparing the permuted distribution to the true mean connectivity for each module, and only modules with p-values < 0.05 were retained. When merging modules from all blocks for the isoform network, a merge cut height of 0.2 was used.

Module eigengenes (MEs) were calculated for all modules using the regressed gene and isoform expression dataset used to generate the networks. We only retained isoform modules that were non-redundant with gene modules forward for further analysis. To achieve this, isoform and gene MEs were clustered using the 'cutTreeHybrid' WGCNA[17] function using average linkage hierarchical clustering of the bi-midweight correlation of the MEs, a deep split of 4, a negative PAMstage, a minimum module size of 1, and a cut height of 0.9999. Any isoform modules that clustered with gene modules were labeled as overlapping with the gene modules, with the exception of Isoform_M26_skyblue3, which upon visual inspection was suitably distant from the other gene modules within its cluster to be considered distinct. To determine if any of these other overlapping isoform modules were distinct enough from the gene modules to be retained for further analysis, for each of the conserved isoform modules a Fisher's exact test was performed with each of the gene modules in its identified cluster. Any isoform modules which had no significant overlap (p > 0.01) were retained for further analysis. In total, 39 distinct isoform modules were carried forward for further analysis out of the original 61 identified isoform modules.

Module functional characterization

We next used linear mixed models to determine whether gene and the distinct isoform module eigengenes were significantly associated with case or control status, controlling for all of the biological covariates from the full models described previously (the technical covariates were not included, since these covariates were previously removed from the regressed expression data used to generate the networks). The same limma[53] workflow was implemented as described before for calculating DE genes and isoforms. Whole cortex and region-specific ASD and dup15q effects were also ascertained as described before for DE gene and isoform analyses. An FDR-adjusted p-value < 0.05 to be considered associated with any ME.

To further functionally characterize modules, we calculated enrichments for neural cell-types, neuronal subtypes, gene ontology terms, protein-protein interactions, the ARI gene groups, gene biotypes (e.g., protein coding, pseudogene, lncRNA, etc, as defined in the Gencode annotation GTF file), relevant GWAS, ASD and epilepsy associated rare variants, and gene modules previously associated with ASD as published [1,5], and described in the following paragraphs.

Neural cell-type enrichment was performed with EWCE as previously described for the ARI gene groups. For neuronal subtype enrichment, medial temporal gyrus single neuron RNA-seq from the Allen Brain Map[23,63] was used to define neuronal subtype specific markers for enrichment analysis with EWCE, because this dataset also contains information on the layer specificity for neuronal subtypes. EWCE was implemented as previously described for the ARI gene groups, with the Allen Brain Map neuronal cells being grouped into cortical layer groups (eg. Exc L2, Inh L2-3), for cell-type enrichment. For gene ontology terms, the Metascape[69] web portal was used with default functions ('Express Analysis'). Only 'GO Biological Process' terms with an FDR-adjusted p-value < 0.05 were examined for each module. PPI annotations and enrichments were calculated with STRING[70] run with default settings. An FDR-corrected PPI enrichment p-value < 0.05 was needed for a module to be considered significantly enriched with PPI. ARI gene group enrichment was calculated using a Fisher's exact test, with an FDR-corrected p-value < 0.05 and OR > 1 being required for a significant enrichment.

Gene biotype enrichment was determined with a permutation approach. The number of each unique gene biotype was first acquired for each module. Then, for each permutation (10,000 in total) gene biotypes were sampled across all genes without replacement and randomly assigned. The number of each unique gene biotype in each module was collected for each permutation. A distribution could then be created for each unique gene biotype in each module across the 10,000 permutations. Both over- and under-enrichment of each unique gene biotype in each module was determined directly with this distribution (one-tailed p-value). An FDR-corrected p-value < 0.05 was required for a significant enrichment.

For the psychiatric GWAS enrichments, partitioned heritability was calculated with stratified LD Score regression[71] (run with recommended settings) using 10 kb windows around genes (matched genes were used for isoform modules). An FDR-corrected p-value < 0.1 was required for a significant GWAS enrichment (the threshold for significance was relaxed since many of the best available GWAS datasets utilized are underpowered, particularly the ASD GWAS. We selected the most recent and best powered GWAS which were relevant and interesting for comparison with these gene and isoform modules, including GWAS conducted for ASD,[21] ADHD,[39] BD,[42] MDD,[43] SCZ,[44] Educational Attainment,[40] Intelligence,[41] and IBD.[45] Logistic regression was used for rare variant enrichment, controlling for both gene length and GC content, with an FDR-corrected p-value < 0.05 being required for a significant enrichment. Syndromic and highly ranked (1 and 2) ASD SFARI gene[22] and high-confidence Epilepsy (compiled by D. Polioudakis et al. Neuron 2019)[72] gene associations were examined. Finally, a Fisher's exact test was used to assess previous module enrichment, with an FDR-corrected p-value < 0.05 and OR >1 indicating a significant positive overlap.

Finally, we assessed the overlap of genes recently published by Jin et al.[73] linked to ASD risk through the Perturb-Seq approach. We used Fisher's exact test to compare Perturb-Seq genes with our DE genes/modules, separating the DE genes from the Perturb-seq analysis (Jin et al. Table S7) into two groups: increased (35 genes) and decreased (11 genes) gene expression changes. Genes were grouped together across cell-types, with any instance of increased or decreased gene expression placing a gene into the 'increased' or 'decreased' groups. We separated the genes into these increased/decreased groups since these opposite effects may signify different biological processes active in ASD pathology. We then conducted one-sided Fisher's Exact Tests to test for significant overlap between the DE genes and gene modules identified in this manuscript. We separated DE genes by increased or decreased expression in ASD, as well as by region-specific or whole cortex detection. For the DE gene tests, we only overlapped Perturb-seq genes with increased expression with DE genes with increased expression and followed a similar approach with the decreased Perturb-seq genes. After correcting for multiple comparisons, we did not see any significant overlap with our DE genes or gene modules (Supplementary Tables 2 and 6, respectively).

Neuronal density and cortical layer 3/4 association with ASD dysregulation

A linear model was used to compare region-specific macaque NeuN density[25] to region-specific ASD effects (model beta) in the regionally-variable gene MEs. Macaque brain areas were matched to Brodmann areas (shared in **Supplementary Data 7**), with six regions matching between this dataset and the macaque dataset. FDR-corrected p-values < 0.1 were considered significant neuronal density associations (the FDR threshold was relaxed, since only 6 regions/points were available for every comparison). A leave-one-out cross-validation was performed to assess individual regional contributions to neuronal density associations, in which a single region was withheld, and linear model statistics were re-calculated. In addition to neuronal density, we also examined the association between cortical layer 4 thickness[28] (von Economo and BigBrain estimates, as shared in the publication[28]) and region-specific ASD effects in the regionally-variable gene MEs. All 11 regions were matched to layer 4 thickness measures (this key is shared in **Supplementary Data 7**). This comparison was also performed with a linear model, with FDR-corrected p-values < 0.05 considered significant for layer 4 thickness associations.

snRNA-seq

Six control and six ASD samples (28 total) matched for covariates (e.g., age, sex, manner of death) were processed in the same nuclear isolation batch to minimize potential batch effects. These subjects included: UMB5144 BA17, BA9,and BA7, AN08792 BA17, BA4-6, and BA7, AN10679 BA17, BA4-6 and BA7, UMB4787 BA17, BA4-6, and BA7, AN19511 BA17, BA4-6, and BA7, UMB4337 BA17, BA4-6, and BA7, AN19760 BA17, BA4-6, and BA7, AN00493 BA17, BA4-6, and BA7, AN07176 BA17, BA4-6, and BA7, UMB5302 BA17, BA9,and BA7, AN15566 BA17, BA9,and BA7, AN12457 AN00493 BA17, BA4-6, and BA7. First, frozen brain samples were placed on dry ice in a dehydrated dissection chamber to reduce degradation effects from sample thawing or humidity then a 50 mg or less section of cortex is taken ensuring specific grey matter /white matter boundary. Tissue was homogenized in 2.5 mL of RNAase-free homogenization buffer (250mM sucrose, 5mM MgCl2, 25mM KCL, 10mM Tris pH8, 1 uM DTT, 0.2U RNaseIN, 1% BSA, 0.01% Triton X-100, 0.001% Digitonin in RNAse-free water) using glass dounce homogenizer on ice. The homogenate was filtered and subjected to a two-layer micro-iodixanol nuclei centrifugal gradient (50%/30%) for 13500g for 20 minutes at 4°C. Supernatant was carefully removed and the nuclei containing pellet were resuspended in RNase-free PBS pH7.4, 5mM MgCl2, 1% BSA, 0.2U RNaseIN. The nuclear suspension was filtered twice through a 30 um cell strainer. Nuclei are inspected for quality (shape, color, membrane integrity) and counted on a countess II machine then loaded onto the 10x Genomics platform to isolation single nuclei and generate libraries. The 10X capture and library preparation protocol was used without modification. Single-nucleus libraries from individual samples were pooled and sequenced on the NovaSeq 6000 machine (average depth 60,000 reads/nucleus).

After sequencing, raw snRNA-seq data processing was performed with 10X Genomics CellRanger software, Seurat (v3.0)[74] and Pegasus (v.1.4.0)[56]. CellRanger was used with default parameters, except we utilized the human pre-mRNA reference file (ENSEMBL GRCh38)[47] to ensure capturing intronic reads originating from pre-mRNA transcripts abundant in the nuclear fraction to generate each library's gene by cell matrices. Pegasus was used to stringently filter cells, remove doublets, integrate and batch-correct all libraries together such that cells with less than 750 genes or above 6,000 genes and more than 10% of their transcriptome is represent by mitochondrial genes are removed. Following filtering and doublet removal, 199,617 cells were kept (out of 254,321) and 34,978 genes (out of 35,412) are kept. Of these 199,617 cells, 113,052 are from occipital cortex, 51,981 are from parietal cortex and 34,584 from the frontal cortex. Each region has closely matched number of ASD to CTL cells represented (occipital cells are broken down into 50,018 cells from ASD cortex and 63,034 cells from the CTL cortex, parietal cells are represented by 29,687 cells from ASD cortex and 22,294 from the CTL cortex, and frontal cells are composed of 18,526 cells from ASD cortex and 16,058 from CTL cortex). Utilizing 65PCs, Harmony (as part of the Pegasus[56] suite) was used to integrate and batch correct libraries, Louvain clustering was performed to cluster cells, plotted on a 2D representation with Uniform

Manifold Approximation and Projection (UMAP). We then visualize integrated cells in two-dimensional space with UMAP.

To gain insight into the regional or diagnostic composition of cell types, cell fractions were calculated for each sample, which then underwent centered-log ratio (clr)-transformation, which accounts for compositional data, and values are interpreted relative to the geometric mean.[58] A repeated-measures ANOVA was used to assess group-level significance, controlling for fixed effects of region, sex, age, library size, nGene, and percent_mito, with a random effect for subject. (**Supplementary Data 8).**

To further explore regional differences in gene expression across diagnosis we performed differential expression analysis using a negative binomial mixed model in the NEBULA R-package[59]. We used model matrix ~Diagnosis+Age+Sex+nGene+percent_mito, where quantitative data are scaled. Filtered raw counts are provided as input and the NEBULA-LN method is used (default values are used for the other options). Significant differentially regulated genes are determined by Benjamini & Hochberg corrected p-values. (**Supplementary Data 8).**

Cell-type deconvolution (CTD)

Methylation Array Based Cell-Type Deconvolution (CTD)

We used single-cell methylome sequencing data from Luo et al.,[29] which profiled 15,030 single cells from post-mortem human frontal cortex tissue from 2 healthy, male donors in their 20s. We generated pseudobulk profiles of excitatory neurons, inhibitory neurons, astrocytes, endothelial cells, microglia, oligodendrocytes and oligodendrocyte precursor cells, with the following filters: 1) restricting to CpG sites on the Illumina 450K array by overlapping with this probe list, after excluding sex chromosomes and MASK probes which have quality issues including cross-hybridization[75]; 2) binning CpG methylation sites within 50bp of an Illumina 450K array CpG site; and 3) taking cytosine sites with >10 read counts.

We identified CpG marker sites using an "extremes" approach. We first converted count data to beta-values (proportion of methylated reads of total read counts). Then, for each of the seven cell-types, we split CpG marker sites into those that were highly-methylated (>=60%) versus those that were lowly-methylated (<=40%). We took marker sites as those that were either highly- or lowly-methylated in a single cell-type, and that were in the opposite methylation "extreme" for all other six cell-types; for example, if a site had <=40% methylation in one cell-type, and had >=60% methylation in all other cell-types. We used the classic Houseman algorithm[76] to estimate cell proportions from bulk methylation. On average, the most prevalent cell-type (by median proportion) was oligodendrocytes, followed by excitatory neurons, inhibitory neurons, astrocytes, microglia, endothelial cells and OPCs. When these cell sub-types were taken in aggregate, glial cells were the dominant cell-type.

Firstly, we tested for global shifts in cell-type proportions by comparing two logistic regression models (Model 0 and Model 1 below) with a likelihood ratio test. Model 1 included extra dependent variables: three principal components that explained >95% of the variance cell-type proportions, using Aitchison principal component analysis, which accounts for compositionality. As we were unable to include individual ID as a random effect due to convergence warnings, we accounted for the correlated standard errors using a robust sandwich variance estimator approach, implemented using the rms:robcov function in R.

$$Model\ 0: ASD \sim age + sex + bank_{brain} + batch + region_{brain}$$
$$Model\ 1: ASD \sim age + sex + bank_{brain} + batch + region_{brain} + PC1_{CTP} + PC2_{CTP} + PC3_{CTP}$$

Model 1 (i.e., including cell-type proportion PCs) had stronger association with ASD diagnosis compared to Model 0 (i.e., excluding cell-type proportion PCs) (chi-squared statistic = 11.69, df = 3, p=8.5e-3). This was primarily related to cell-type proportion PC2, which represented higher loadings of excitatory neurons and oligodendrocytes, and decreased microglia (**Supplementary Data 7**).

Secondly, to quantify the effect sizes of each cell-type on ASD diagnosis, we performed multivariate linear regression to regress centred-log ratio (clr)-transformed cell-type proportions (offset=1e-3) against ASD diagnosis (covariates: age, sex, brain bank, batch). The clr-transformation accounts for compositional data, and values are interpreted relative to the geometric mean.[58] Only increased microglia in the prefrontal cortex and decreased oligodendrocytes lobe had nominal association with ASD diagnosis after accounting for covariates. Overall, there was evidence of global cell-type compositional shifts, but little evidence of individual cell-type proportion shifts in ASD case versus control brains, after FDR correction (**Supplementary Data 7**).

Supplementary References

61. earth: Multivariate Adaptive Regression Splines. *Comprehensive R Archive Network (CRAN)* http://CRAN.R-project.org/package=earth.

62. Hoffman, G. E. & Schadt, E. E. variancePartition: interpreting drivers of variation in complex gene expression studies. *BMC Bioinformatics* **17**, 483 (2016).

63. Skene, N. G. & Grant, S. G. N. Identification of Vulnerable Cell Types in Major Brain Disorders Using Single Cell Transcriptomes and Expression Weighted Cell Type Enrichment. *Front. Neurosci.* **10**, 16 (2016).

64. Lake, B. B. *et al.* Integrative single-cell analysis of transcriptional and epigenetic states in the human adult brain. *Nat. Biotechnol.* **36**, 70–80 (2018).

65. Reimand, J., Arak, T. & Vilo, J. g:Profiler—a web server for functional interpretation of gene lists (2011 update). *Nucleic Acids Res.* **39**, W307–W315 (2011).

66. Carbon, S. *et al.* AmiGO: online access to ontology and annotation data. *Bioinformatics* **25**, 288–289 (2009).

67. Ashburner, M. *et al.* Gene ontology: tool for the unification of biology. The Gene Ontology Consortium. *Nat. Genet.* **25**, 25–29 (2000).

68. The Gene Ontology Consortium. The Gene Ontology Resource: 20 years and still GOing strong. *Nucleic Acids Res.* **47**, D330–D338 (2019).

69. Zhou, Y. *et al.* Metascape provides a biologist-oriented resource for the analysis of systems-level datasets. *Nat. Commun.* **10**, 1523 (2019).

70. Szklarczyk, D. *et al.* The STRING database in 2021: customizable protein-protein networks, and functional characterization of user-uploaded gene/measurement sets. *Nucleic Acids Res.* **49**, D605–D612 (2021).

71. Finucane, H. K. *et al.* Partitioning heritability by functional annotation using genome-wide association summary statistics. *Nat. Genet.* **47**, 1228–1235 (2015).

72. Polioudakis, D. et al. A Single-Cell Transcriptomic Atlas of Human Neocortical Development during Mid- gestation. Neuron 103, 785-801.e8 (2019).

73. Jin, X. *et al.* In vivo Perturb-Seq reveals neuronal and glial abnormalities associated with autism risk genes. *Science* **370**, (2020).

74. Butler, A., Hoffman, P., Smibert, P., Papalexi, E. & Satija, R. Integrating single-cell transcriptomic data across different conditions, technologies, and species. *Nat. Biotechnol.* **36**, 411–420 (2018)

75. Zhou, W., Laird, P. W. & Shen, H. Comprehensive characterization, annotation and innovative use of Infinium DNA methylation BeadChip probes. *Nucleic Acids Res*. **45**, e22 (2017)

76. Houseman, E. A. et al. DNA methylation arrays as surrogate measures of cell mixture distribution. *BMC Bioinformatics* **13**, 86 (2012).
