# Supplementary material for: Broad transcriptomic dysregulation occurs across the cerebral cortex in ASD
Source: Nature. 2022 Nov 2;611(7936):532–9. doi: 10.1038/s41586-022-05377-7 (PMC9668748; doi:10.1038/s41586-022-05377-7)
Supplement: Supplementary file 2 — Reporting Summary [file 41586_2022_5377_MOESM2_ESM.pdf]

nature portfolio

Corresponding author(s): Gandal, Geschwind

Last updated by author(s): Jul 8, 2022

Reporting Summary

Nature Portfolio wishes to improve the reproducibility of the work that we publish. This form provides structure for consistency and transparency in reporting. For further information on Nature Portfolio policies, see our Editorial Policies and the Editorial Policy Checklist.

Statistics

For all statistical analyses, confirm that the following items are present in the figure legend, table legend, main text, or Methods section.

n/a	Confirmed	
☐	☒	The exact sample size (*n*) for each experimental group/condition, given as a discrete number and unit of measurement
☐	☒	A statement on whether measurements were taken from distinct samples or whether the same sample was measured repeatedly
☐	☒	The statistical test(s) used AND whether they are one- or two-sided<br>*Only common tests should be described solely by name; describe more complex techniques in the Methods section.*
☐	☒	A description of all covariates tested
☐	☒	A description of any assumptions or corrections, such as tests of normality and adjustment for multiple comparisons
☐	☒	A full description of the statistical parameters including central tendency (e.g. means) or other basic estimates (e.g. regression coefficient) AND variation (e.g. standard deviation) or associated estimates of uncertainty (e.g. confidence intervals)
☐	☒	For null hypothesis testing, the test statistic (e.g. *F*, *t*, *r*) with confidence intervals, effect sizes, degrees of freedom and *P* value noted<br>*Give P values as exact values whenever suitable.*
☒	☐	For Bayesian analysis, information on the choice of priors and Markov chain Monte Carlo settings
☒	☐	For hierarchical and complex designs, identification of the appropriate level for tests and full reporting of outcomes
☐	☒	Estimates of effect sizes (e.g. Cohen's *d*, Pearson's *r*), indicating how they were calculated

Our web collection on statistics for biologists contains articles on many of the points above.

Software and code

Policy information about availability of computer code

Data collection	No software was used for data collection
Data analysis	All analyses were conducted in R (version 3.4 or higher). All software used is described in the Methods and Supplementary methods. Analysis code used is available at https://github.com/dhglab/Broad-transcriptomic-dysregulation-across-the-cerebral-cortex-in-ASD. Bulk RNA-seq data quality control was conducted using FastQC (v0.11.2), STAR (v2.5.2b), PicardTools (v2.5.0), VerifyBAMID (v1.1.3), and the EARTH package (v5.3.0). Alignment was performed with STAR as above using the GRCh37.p13 genome build and Gencode v25lift37 annotations. Gene and transcript-isoform expression quantification was performed using RSEM (v1.3.0). Differential expression was conducted with limma (3.46), network analysis using WGCNA (v1.68), pathway analysis with gProfileR (0.70). Analysis of single-nucleus RNAseq data was conducted with the 10X Cell Ranger cloud software (v6), Seurat (v3.0), the Pegasus suite (v1.4.0), and the NEBULA R package (v1.2.0).

For manuscripts utilizing custom algorithms or software that are central to the research but not yet described in published literature, software must be made available to editors and reviewers. We strongly encourage code deposition in a community repository (e.g. GitHub). See the Nature Portfolio guidelines for submitting code & software for further information.

Data

Policy information about availability of data

All manuscripts must include a data availability statement. This statement should provide the following information, where applicable:

- Accession codes, unique identifiers, or web links for publicly available datasets
- A description of any restrictions on data availability
- For clinical datasets or third party data, please ensure that the statement adheres to our policy

The source data (bulk and snRNA-seq) generated in this manuscript are available via the PsychENCODE Knowledge Portal (https://psychencode.synapse.org/) with the doi: doi.org/10.7303/syn4587615. The PsychENCODE Knowledge Portal is a platform for accessing data, analyses, and tools generated through grants funded by the National Institute of Mental Health (NIMH) PsychENCODE program. Data is available for general research use according to the following requirements for data access and data attribution: (https://psychencode.synapse.org/DataAccess). Single cell and bulk RNAseq data from the Allen Brain Atlas were downloaded from http://portal.brain-map.org/. Single cell methylation data from Luo et al is available on GEO (GSE140493).

Human research participants

Policy information about studies involving human research participants and Sex and Gender in Research.

Reporting on sex and gender	Sex was included as a covariate in all statistical analyses. As ASD predominately affects males (~4:1), case and control groups were balanced by sex.
Population characteristics	Population characteristics were individually collected and provided by each brain bank using available demographic and clinical data. Available data is included in Supplementary Table 1.
Recruitment	Postmortem cortical brain samples were acquired from the Harvard Brain Bank as part of the Autism BrainNet project (formerly the Autism Tissue Project, ATP) and the University of Maryland Brain Banks (UMDB). Sample recruitment acquisition protocols were followed for each brain bank. Samples were de-identified before acquisition for this study, which was not directly involved in any recruitment process.
Ethics oversight	Postmortem samples were de-identified before acquisition and thus exempt from IRB review.

Note that full information on the approval of the study protocol must also be provided in the manuscript.

Field-specific reporting

Please select the one below that is the best fit for your research. If you are not sure, read the appropriate sections before making your selection.

☒ Life sciences ☐ Behavioural & social sciences ☐ Ecological, evolutionary & environmental sciences

For a reference copy of the document with all sections, see nature.com/documents/nr-reporting-summary-flat.pdf

Life sciences study design

All studies must disclose on these points even when the disclosure is negative.

Sample size	Sample sizes were chosen based on (1) sample availability and (2) power analyses with previously analyzed samples (as published in Parikshak et al., Nature 2016).
Data exclusions	Outliers were removed from main analyses that based on unsupervised clustering (principal component analysis) of sample network connectivity measures (described in the Methods). This was done in accordance with our previously published work (Parikshak et al., 2016; Gandal et al., 2018a) to mitigate the potential influence of biological or sequencing-related technical biases that may have affected a small number of samples. These outliers are still included with the raw data.
Replication	Bootstraping analyses (subsampling 1000 times with replacement) were conducted to validate results as described in the supplementary materials. For the transcriptomic regional identity analysis, the Allen Brain Atlas was used to replicate results (for matching regions). Replication was further evaluated by comparing results from idiopathic ASD samples compared with the dup15q cohort, which showed concordant alterations in differential expression and transcriptomic regional identity. No other dataset exists, to our knowledge, that we can use to externally validate our complete results.
Randomization	Randomization is not relevant here - we obtained samples (with blinded identities) from brain banks (see Methods), and were given specific biological information about all samples (ASD status, age, etc.). Within our covariate groups of interest, when subsets of samples were extracted for certain analyses, these subsets were random.
Blinding	Blinding is not relevant here - we obtained samples (with blinded identities) from brain banks (see Methods), and were given specific biological information about all samples (ASD status, age, etc.).

Reporting for specific materials, systems and methods

We require information from authors about some types of materials, experimental systems and methods used in many studies. Here, indicate whether each material, system or method listed is relevant to your study. If you are not sure if a list item applies to your research, read the appropriate section before selecting a response.

Materials & experimental systems

n/a	Involved in the study
☒	☐ Antibodies
☒	☐ Eukaryotic cell lines
☒	☐ Palaeontology and archaeology
☒	☐ Animals and other organisms
☒	☐ Clinical data
☒	☐ Dual use research of concern

Methods

n/a	Involved in the study
☒	☐ ChIP-seq
☒	☐ Flow cytometry
☒	☐ MRI-based neuroimaging
